# Reversible hydrogen control of antiferromagnetic anisotropy in α-Fe_2_O_3_

**DOI:** 10.1038/s41467-021-21807-y

**Published:** 2021-03-12

**Authors:** Hariom Jani, Jiajun Linghu, Sonu Hooda, Rajesh V. Chopdekar, Changjian Li, Ganesh Ji Omar, Saurav Prakash, Yonghua Du, Ping Yang, Agnieszka Banas, Krzysztof Banas, Siddhartha Ghosh, Sunil Ojha, G. R. Umapathy, Dinakar Kanjilal, A. Ariando, Stephen J. Pennycook, Elke Arenholz, Paolo G. Radaelli, J. M. D. Coey, Yuan Ping Feng, T. Venkatesan

**Affiliations:** 1 NUSNNI-NanoCore, National University of Singapore, Singapore, Singapore; 2 NUS Graduate School - Integrative Sciences and Engineering Programme, National University of Singapore, Singapore, Singapore; 3 Department of Physics, National University of Singapore, Singapore, Singapore; 4 Advanced Light Source, Lawrence Berkeley National Laboratory, Berkeley, CA USA; 5 Department of Materials Science and Engineering, National University of Singapore, Singapore, Singapore; 6 Institute of Chemical and Engineering Sciences, Singapore, Singapore; 7 Singapore Synchrotron Light Source, National University of Singapore, Singapore, Singapore; 8 Inter-University Accelerator Centre, New Delhi, India; 9 Clarendon Laboratory, Department of Physics, University of Oxford, Oxford, UK; 10 School of Physics, Trinity College, Dublin, Ireland; 11 CRANN, Trinity College, Dublin, Ireland; 12 Department of Electrical and Computer Engineering, National University of Singapore, Singapore, Singapore; 13 Present Address: Chang’an University, Xi’an, China; 14 Present Address: National Synchrotron Light Source II, Upton, NY USA; 15 Present Address: Department of Physics, SRM University - AP, Amaravati, Andhra Pradesh India; 16 Present Address: Cornell High Energy Synchrotron Source, Ithaca, NY USA

**Keywords:** Magnetic properties and materials, Spintronics, Magnetic properties and materials, Phase transitions and critical phenomena, Surfaces, interfaces and thin films

## Abstract

Antiferromagnetic insulators are a ubiquitous class of magnetic materials, holding the promise of low-dissipation spin-based computing devices that can display ultra-fast switching and are robust against stray fields. However, their imperviousness to magnetic fields also makes them difficult to control in a reversible and scalable manner. Here we demonstrate a novel proof-of-principle ionic approach to control the spin reorientation (Morin) transition reversibly in the common antiferromagnetic insulator α-Fe_2_O_3_ (haematite) – now an emerging spintronic material that hosts topological antiferromagnetic spin-textures and long magnon-diffusion lengths. We use a low-temperature catalytic-spillover process involving the post-growth incorporation or removal of hydrogen from α-Fe_2_O_3_ thin films. Hydrogenation drives pronounced changes in its magnetic anisotropy, Néel vector orientation and canted magnetism via electron injection and local distortions. We explain these effects with a detailed magnetic anisotropy model and first-principles calculations. Tailoring our work for future applications, we demonstrate reversible control of the room-temperature spin-state by doping/expelling hydrogen in Rh-substituted α-Fe_2_O_3_.

## Abstract (web summary)

One major challenge for antiferromagnetic spintronics is how to control the antiferromagnetic state. Here Jani et al. demonstrate the reversible ionic control of the room-temperature magnetic anisotropy and spin reorientation transition in haematite, via the incorporation and removal of hydrogen.

## Introduction

Antiferromagnets (AFM) exhibit long-range magnetic order with two or more sublattices aligned so that they produce no net magnetization. There has recently been a surge of interest^1–4^ as AFMs have the potential to replace ferromagnets in various spin-based devices. Among them, insulators promise efficient magnon-based spin transport with the absence of charge-related Joule losses^5,6^. They also host attractive functionalities like spin colossal-magnetoresistance^7^, ultra-fast dynamics^8–11^, high domain-wall propagation velocities^12,13^ and spin-superfluidity^14^. Despite all these advantages, they have so far played only an auxiliary role as pinning or exchange bias layers in thin film spintronic devices as their magnetic order is difficult to detect and hard to control^1,15^. Although many recent reports demonstrating clear detection of the AFM-state in insulators by spin-Hall or anisotropic magneto-transport have emerged^7,16–18^, control of the ground-state continues to be a challenge^15^.

In this work, we focus on α-Fe_2_O_3_, which is a well-known antiferromagnetic insulator with a bulk Dzyaloshinskii–Moriya interaction (DMI) that undergoes a spin-reorientation Morin transition between the in-plane and out-of-plane states (at $T_{\mathrm{M}_{\mathrm{bulk}}}$ ~ 260 K)^19^. Controlling antiferromagnetism in α-Fe_2_O_3_ is important as it would open prospects for both magnonics and real-space topological spintronics. This is because the material exhibits ultra-low Gilbert damping^6,20^, has exceptionally-long and tunable spin diffusion (in the microns range)^6,20,21^, sizable spin-Hall magnetoresistance^16,18^ (when combined with a Pt overlayer) and is also the only reported natural AFM to date that hosts a wide family of topological AFM textures^22–24^ at room temperature. Consequently, reversible engineering of the anisotropy, and thereby the Morin transition, is crucial as it would allow control over magnon-transport^20,21^ and topological texture dimensions^22^. Previous reports have shown that strain^25^, chemical doping^19,26–30^ and meso-structure^31^ can modify the antiferromagnetism in α-Fe_2_O_3_. However, lack of reversibility and scalability makes these approaches difficult to exploit in practical applications.

In the literature, coherent optical techniques have enjoyed success in manipulating antiferromagnetic insulators^8,9^, but they require table-top lasers and large interaction volumes. Secondly, electric-field control of the AFM-state, which is possible in multiferroic^32^ and magnetoelectric^3,33^ oxides, cannot be replicated in a vast majority of functional materials, where magnetic and electrical degrees of freedom are not coupled. Alternatively, electric-field control via strain coupling to piezoelectric substrates, which has been quite successful in tuning antiferromagnetic metals^34–36^, require epitaxial matching or texturing constraints to be satisfied for crystalline systems^36,37^. Thirdly, electric-current control of the AFM-state via Néel spin–orbit torques is also quite useful^2,38,39^, but is limited to materials with strong spin–orbit coupling that break local inversion-symmetry in a specific manner. Finally, spin-Hall effect based spin–orbit torques injected from a heavy-metal overlayer are also quite promising in controlling the AFM-state of insulators^40–43^. However, studies in α-Fe_2_O_3_ have so far shown in-plane tuning^42,43^, which is insufficient to directly control the Morin transition. Hence, there is a need for developing alternative pathways to directly tune antiferromagnetic properties and spin reorientation in α-Fe_2_O_3_ thin films.

Here, we present a new method of hydrogen (H)-doping to tailor antiferromagnetism in epitaxial α-Fe_2_O_3_ thin films by using spillover. We draw inspiration from pioneering ionic studies, where doping light ions (e.g. H, Li, O etc.) was used to control anisotropy in ferromagnetic systems^44,45^, or transport in strongly correlated-oxides^46–48^ and conventional insulators^49^, although the crystal physics and chemistry underpinning our implementation is completely different. By incorporating or removing hydrogen, we demonstrate a hitherto unexplored ionic approach to control a wide range of antiferromagnetic properties in a reversible and stable manner. H-doping can drive these pronounced changes by delicately tuning the competing contributions to the magnetic anisotropy, as a result of local electron injection and ensuing local distortions. Lastly, we show reversible control of the room-temperature Morin transition by hydrogenation of α-Fe_1.97_Rh_0.03_O_3_ films.

## Results and discussion

**Fig. 1:**
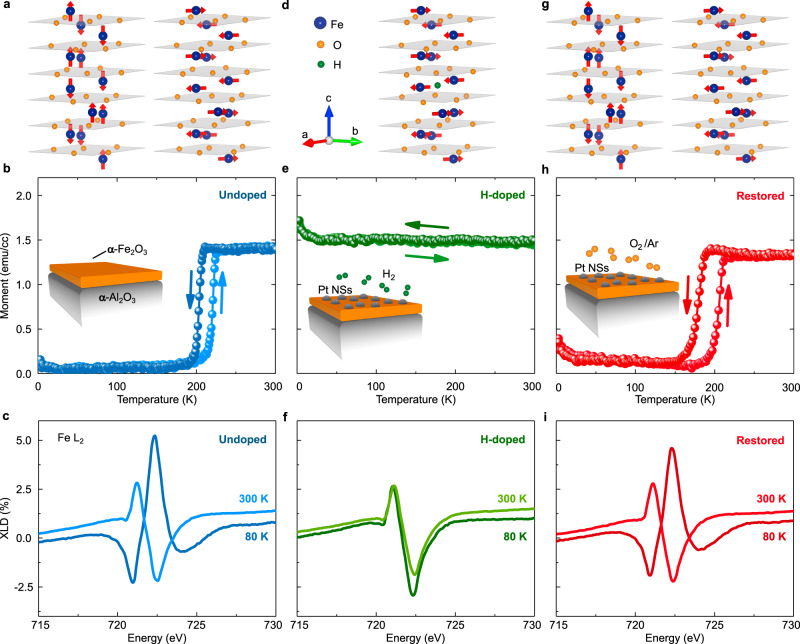
Reversible control of the antiferromagnetic Néel vector orientation and the Morin transition by H-doping. **a** Schematic of α-Fe_2_O_3_ crystal unit cell and the spin alignment at $T \,< \,T_{{\mathrm{M}}_0}$ (left) and $T \,> \,T_{{\mathrm{M}}_0}$ (right). **b** *M*(*T*) curve of undoped α-Fe_2_O_3_ films during up/down temperature sweeps in a measuring field of 500 Oe (following field cooling at 500 Oe), exhibited a transition with the appearance/disappearance of the in-plane canted moment. **c** Fe L_2_ edge XLD spectra measured below (80 K) and above (300 K) $T_{{\mathrm{M}}_0}$, showed opposite signatures, validating the anisotropy switch in (**a**). **d**–**f** Unit cell of H-doped α-Fe_2_O_3_ with in-plane **L** orientation throughout the temperature range (**d**), deduced from the transition-less *M*(*T*) curve (**e**), and XLD showing the same asymmetry at 80 K and 300 K (**f**). **g**–**i** Unit cell of H-doped α-Fe_2_O_3_ sample after restoration step in 100% O_2_ atmosphere, exhibited the out-of-plane to in-plane **L** transition (**g**), inferred from the return of the Morin transition in *M*(*T*) (**h**), and XLD showing asymmetry reversal (**i**). The restored state can also be obtained by annealing the H-doped films in 100% Ar atmosphere (see Supplementary-S5). Insets in (**b**, **e**, **h**) correspond to the sample structure of **b** undoped, **e** H-doped, **h** restored α-Fe_2_O_3_ films in corresponding environments. The discontinuous Pt nano-structures (NSs) are also indicated.

α-Fe_2_O_3_ has the trigonal corundum structure, with antiparallel sublattices (with magnetizations **M**_1,2_) stacked alternately along the hexagonal *c*-axis, as shown in Fig. 1a. At room temperature, Fe spins lie in the basal planes with the Néel vector, ${\mathbf{L}} \equiv {\mathbf{M}}_1 - {\mathbf{M}}_2$, perpendicular to the *c*-axis. Presence of the bulk DMI causes canting of the sublattices within the basal planes^19^, resulting in a weak macroscopic in-plane canting (Supplementary-S1). Upon cooling, α-Fe_2_O_3_ undergoes the Morin transition^19,26^, where the sublattices reorient to the *c*-axis—a Néel-state where the canted moment ideally vanishes by symmetry. The Morin transition results from a delicate temperature-controlled crossover between magnetic-dipolar and single-ion magnetic anisotropy contributions^50^.

### Reversible spin reorientation by H-doping

Highly-oriented epitaxial thin films of α-Fe_2_O_3_ were grown on (0001)-oriented α-Al_2_O_3_ single crystals by pulsed laser deposition (‘Methods’, Supplementary-S2). These films exhibited a clear Morin transition in temperature-dependent magnetometry, *M(T)*, with hysteretic appearance/disappearance of the in-plane canted moment, Fig. 1b. The $T_{{\mathrm{M}}_0}$, defined as the Morin transition temperature of the as-grown sample prior to H-doping, is lower than the bulk value $T_{\mathrm{M}_{\mathrm{bulk}}}$ because of substrate strain^25^. To confirm the magnetic origin of the transition, we performed X-ray linear dichroism (XLD) spectroscopy at the Fe L_2_ edge, which is a good element-specific approach to identify the direction of the sublattice magnetization in α-Fe_2_O_3_ (‘Methods’)^51^. The dichroism reverses sign across $T_{{\mathrm{M}}_0}$ (Fig. 1c), due to the 90° reorientation of **L** to the basal plane from the *c*-axis. We also performed X-ray magnetic circular dichroism (XMCD) spectroscopy but saw no signal from the weak canted moment (Supplementary-S6). Field-dependent magnetometry, *M*(*H*), revealed a hysteretic evolution due to in-plane realignment of antiferromagnetic domains driven by the magnetic field coupling to the canted moment, at $T \,> \,T_{{\mathrm{M}}_0}$ (Supplementary-S10). The hysteretic response significantly suppresses  when $T \,< \,T_{{\mathrm{M}}_0}$. These results establish that the magnetization signals in our samples are intrinsic, and do not arise from any spurious ferro- or ferrimagnetic iron-oxide phases.

To achieve hydrogenation of α-Fe_2_O_3_, catalytic spillover^46,52,53^ of hydrogen into our films was performed through sputtered platinum nano-structures (Fig. 1e inset, ‘Methods’ and Supplementary-S3). Films were annealed in forming gas (H_2_/Ar ratio of 5%/95%) at low temperatures (150–270 °C, Supplementary-S4). In this process, H_2_ gas dissociates into reactive H atoms at the gas-catalyst-oxide triple-phase boundary^46,52^, and protons and electrons together enter the film. The protons then diffuse in the bulk of the oxide and have a propensity to bond with O-anions to form OH^−^, while the electrons are added to the *d*-shells of neighbouring Fe cations, causing local valence-reduction. To confirm that hydrogen is incorporated into our films, elastic recoil detection analysis (ERDA) was used (see ‘Methods’). The hydrogen concentration in the H-doped films was found to be in the range ~1.57 ± 0.14 at.% to 2.47 ± 0.14 at.% depending on the H-spillover temperature (Supplementary-S7). One plausible hypothesis relating spillover temperature to H-concentration is the modification of bulk-diffusion  via thermodynamic activation ^54^. By contrast, oxygen resonant Rutherford backscattering spectrometry (RRBS) performed in tandem with ERDA, which is resonantly sensitive to the O-content of films (rather than O-content of the substrate, Supplementary-S7), revealed that Fe–O stoichiometry remains similar after H incorporation. Structural studies performed by high-resolution X-ray diffraction (HR-XRD), reciprocal space mapping (RSM) and high-angle annular dark-field scanning transmission electron microscopy (HAADF-STEM) showed that our catalytic approach slightly expands the lattice constants, while leaving the film coherence and crystal structure effectively unchanged (see Supplementary-S8). This result distinguishes our catalytic method from other high-temperature hydrogenation experiments^55^, which completely changed the oxide phase (Supplementary-S4). In particular, absence of resonant features in Fe L XMCD or spurious superparamagnetic or ferrimagnetic signals further suggests that the presence of magnetite in the H-doped samples should be negligible (see Supplementary-S6, S10).

Magnetometry of the H-doped samples prepared by spillover at 250 °C showed no Morin transition down to cryogenic temperatures (Fig. 1e). The XLD of hydrogenated samples at room and low temperatures exhibited similar Fe L_2_ dichroic signatures (Fig. 1f), suggesting that the film retains the in-plane orientation of the Néel vector—**L**, at cryogenic temperatures. Hence, a modest quantity of hydrogen supresses the Morin transition, producing a 90° reorientation of **L** from the Néel-state to the canted-state at all $T \,< \,T_{{\mathrm{M}}_0}$. We find that the hydrogenation-driven transformation is quite stable, with retention of the canted-state for at least a year (Supplementary-S4).

**Fig. 2:**
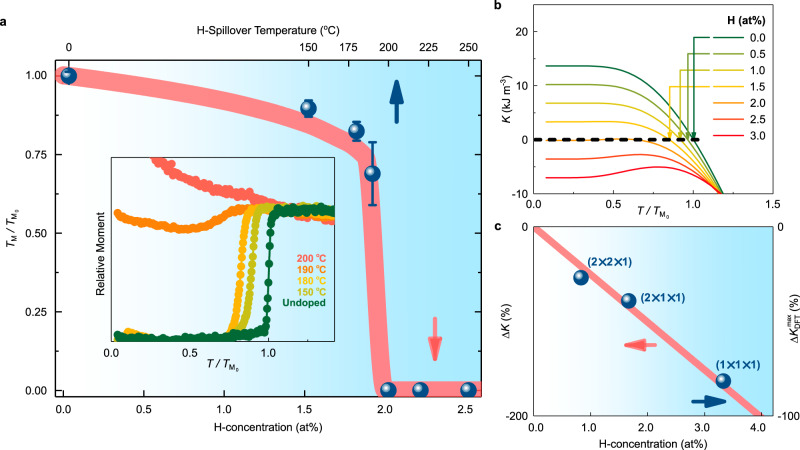
Temperature and doping evolution of the Morin transition and overall magnetic anisotropy. **a** Evolution of the ratio of the Morin transitions after and before H-doping ($T_{\mathrm{M}}/T_{{\mathrm{M}}_0}\!$) in α-Fe_2_O_3_ films, as a function of the H-spillover temperature (upper abscissa), shown as blue spheres. Error bars represent the width of the transition. H-spillover temperature is linked to the H-concentration (lower abscissa) from ERDA results (see Supplementary-S7). The blue shading increases with H-concentration. Inset shows the relative moment of some of the H annealed samples measured during temperature up-sweep in a measuring field of 500 Oe (following cooling at 5000 Oe). **b** Temperature-dependent trends of the total anisotropy at various fixed values of H (at.%), i.e. at fixed Fe^2+^-cation concentration, $K(x = {\mathrm{const}},T)$, obtained from our phenomenological model. Positive and negative values of *K* stabilize out-of-plane and in-plane orientations, respectively. The Morin transition temperature as a function of doping, $T_{\mathrm{M}}(x)/T_{{\mathrm{M}}_0}$, obtained by solving $K\left( {x = {\mathrm{const}},T_{\mathrm{M}}} \right) = 0$, is plotted as the red line in the main panel of (**a**). **c** Evolution of the total anisotropy change as a function of H-doping at 0 K, ${{\Delta }}K\left( {x,T = 0} \right)$, obtained as a percentage from the phenomenological model is given as the red line (left ordinate). The DFT calculated maximum value of the total anisotropy change, ${{\Delta }}K_{{\mathrm{DFT}}}^{{\mathrm{max}}}(x)$, for *H*_i_ containing α-Fe_2_O_3_ cells of three sizes is represented as blue spheres (right ordinate).

Furthermore, the H-concentration in the oxide can be modified by the choice of spillover temperature (Supplementary-S7). At low spillover temperatures (~150–180 °C, see Fig. 2a inset), instead of complete suppression of the Morin transition, we observed its gradual reduction with respect to the undoped counterpart, $T_{{\mathrm{M}}_0}$. This modified Morin temperature after H-spillover is termed as $T_{\mathrm{M}}$, differently from $T_{{\mathrm{M}}_0}$, and $T_{\mathrm{M}}/T_{{\mathrm{M}}_0}$ is displayed in Fig. 2a (upper abscissa). At intermediate spillover temperatures both $T_{\mathrm{M}}$ and the transition height of the canted moment were reduced, presumably due to the presence of small remnant in-plane fractions at low temperatures, as is well-known in the α-Fe_2_O_3_ doping literature^28,29^. Finally, above a threshold spillover temperature value of ~200 °C, the Morin transition, and therefore $T_{\mathrm{M}}/T_{{\mathrm{M}}_0}$, was suppressed completely. Moreover, *M*(*H*) curves of the H-doped films revealed a multi-hysteretic in-plane evolution of the antiferromagnetic domains possibly due to the enhancement of local DMI after H incorporation (Supplementary-S10).

Interestingly, the effects of H-spillover can be reversed by annealing the H-doped samples in 100% Ar or O_2_ atmospheres, which helps to de-hydrogenate^46,56^ the films and switch the magnetic anisotropy back again by substantially recovering the original transition (Fig. 1g–i). In particular, H-doped α-Fe_2_O_3_ samples annealed in a 100% Ar atmosphere also recovered the Morin transition (Supplementary-S5), strongly suggesting that the role of O-vacancies is minimal, and that the observed effects emerge principally from H incorporation and removal. Finally, control experiments of undoped α-Fe_2_O_3_ samples annealed in 100% Ar atmosphere (without any H_2_) had little effect on the Morin transition (Supplementary-S5), demonstrating that annealing α-Fe_2_O_3_ in Ar-rich, and thereby oxygen-poor atmosphere, fails to reproduce the anisotropy changes driven by H-doping.

Overall, these results show that the Néel vector orientation and the magnetic anisotropy can be toggled reversibly by the incorporation/removal of a small amount of hydrogen into the host lattice, without introducing significant structural changes or deleterious phase transitions. This feature is important for potentially exploiting ionic pathways in low-energy spintronic implementations.

### Electron injection driven by H-doping

**Fig. 3:**
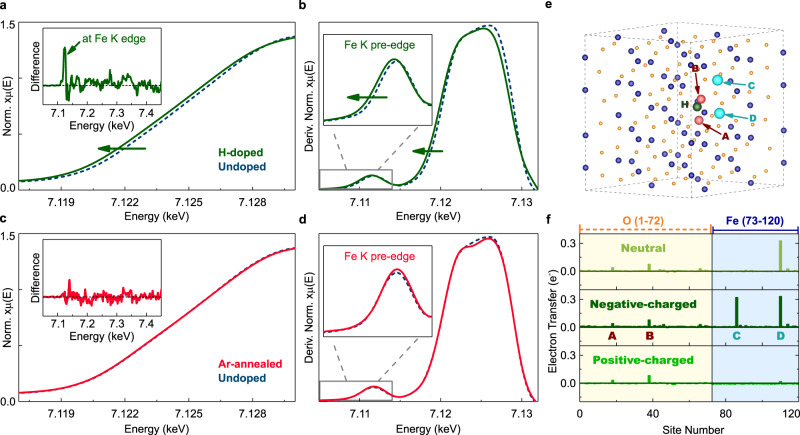
Localised electron injection at Fe cations driven by H-doping. **a**, **b** Normalized Fe K edge XANES spectra (**a**) and their derivatives (**b**) of the H-doped (green solid line) and undoped (blue dashed line) α-Fe_2_O_3_ films, respectively. The main-edge of H-doped samples is red-shifted relative to the undoped counterparts (see Supplementary-S9 for complete Fe K edge spectrum). Inset in (**a**) shows difference spectrum (H-doped – Undoped) with a sharp positive peak at the near-edge and negligible changes in the extended-edge regions. Inset in (**b**) shows a red-shift of the pre-edge shoulder after H-doping. **c**, **d** Normalized XANES spectra (**c**) and their derivatives (**d**) of the Ar-annealed (red solid line) control and undoped α-Fe_2_O_3_ films, respectively. Spectrum of the Ar-annealed samples shows no energy shift relative to the undoped counterpart. Inset in (**c**) shows the difference spectrum (Ar-annealed – Undoped) with negligible changes in all regions. Inset in (**d**) reveals the absence of a shift in the pre-edge shoulder. **e** 2 × 2 × 1 hexagonal super-cell of α-Fe_2_O_3_ with Fe (blue) and O (orange) atoms (120 in total). Specific Fe or O-sites demarcated as C/D (cyan) or A/B (red), respectively, that receive significant fraction of the Bader-charge from the interstitial H-dopant (green). **f** Effective Bader-charge transferred to the 120 atoms obtained from DFT calculations after addition of H-dopant in the super-cell. Here positive and negative peaks correspond to electron injection to and withdrawal from that site, respectively. Neutral and charge-doped regimes of α-Fe_2_O_3_ are obtained by modifying the quantum of the excess electronic charge added to the super-cell (see ‘Methods’ and Supplementary-S15). The O and Fe sites are labelled 1–72 and 73–120, respectively.

To understand the effect of H-doping we performed X-ray absorption near-edge structure (XANES) in a bulk-sensitive fluorescence mode at the Fe K edge. A systematic chemical shift of the Fe K edge and shoulder to lower binding energies was evident in the normalized spectrum and its derivative, of the H-doped samples with respect to the undoped counterparts (Fig. 3a, b). However, in the extended-edge region, the spectra only differed slightly (Supplementary-S9). This suggests that hydrogenation leads to electron injection at Fe^3+^-cation sites, without inducing large average changes in the Fe–O molecular bonding framework. For H-doped films (prepared at 250 °C, Fig. 1e, f), the fraction of reduced Fe species was estimated to be ~2.4 ± 0.5 at.% (see Supplementary-S9), which closely matched the H-concentration observed by ERDA (~2.47 ± 0.14 at.%). This observation supports the role of H-dopants as electron donors, consistent with previous reports in other oxides, which also exhibit chemical red-shifts concomitant with H-doping^46,47,52^. By contrast, XAS spectrum of control undoped samples annealed in 100% Ar did not exhibit any chemical shifts at the near- and extended-edge regions (Fig. 3c, d), unequivocally establishing that it is not an O-deficient condition but the H incorporation from spillover that is responsible for electron injection.

To understand this charge transfer’s origin, we performed density functional theory (DFT) calculations of defect formation and charge transfer in a 2 × 2 × 1 super-cell (hexagonal) of α-Fe_2_O_3_ containing different types of point defects (interstitial H—*H*_i_; substitutional-H occupying an O-vacancy site—*H*_O_; or O-vacancy—*V*_O_), see ‘Methods’. In addition, we manually introduced or removed electrons to or from this defect-containing super-cell, to mimic charge-doped regimes of *α*-Fe_2_O_3_. We found, firstly, that H-dopants prefer to be interstitials, with the formation energy of *H*_i_ markedly lower than *H*_O_/*V*_O_ in all charge-doped regimes (see Supplementary-S15). This underscores the negligible role of the latter defects and primary role of the former. Further, these *H*_i_ are theoretically predicted to form O–H bonds in the lattice, which were experimentally confirmed via Fourier transform infra-red (FTIR) spectroscopy of H-doped α-Fe_2_O_3_ (see Supplementary-S8). Consequently, incorporation of *H*_i_ results in small non-uniform local distortions of the Fe–O bonding network, manifesting as localized Fe–O bond-length deviations as well as FeO_6_ octahedral distortions in our DFT calculations (see Supplementary-S16).

Secondly, we calculated the Bader-charge, employing zero-flux surfaces to partition the electronic charge in a super-cell, to understand charge redistribution upon H-doping. The change of the Bader-charge at every atom after addition of *H*_i_ corresponds to the *H*_i_-driven charge transfer. In the neutral and negative-charged cases, we observed that only a small fraction of Bader-charge is transferred from *H*_i_ to the nearby O-sites (labelled A, B in Fig. 3e) to form OH bonds, while most of the Bader-charge is donated to the Fe sites (labelled C, D) to form locally reduced Fe cations, see Fig. 3f. In the positive-charged condition, OH bond still exists, while the Bader-charge transferred to Fe sites presumably compensates the cationic-charge withdrawn due to acceptors. Experimentally, the negative-charged/neutral regimes are most relevant for our films, as high-temperature growth conditions could cause extremely trace amounts of electron-donating O-vacancies. Hence, according to Fig. 3f (upper and central panels), we conclude that the electrons donated by H-dopants in α-Fe_2_O_3_ should be centred at Fe sites in the immediate vicinity of *H*_i_. This suggests that the charge transfer observed in our XANES red-shifts (Fig. 3a, b) actually corresponds to localized excess charge transferred to the Fe cations, after H-doping. This is reasonable given that any excess electrons donated in α-Fe_2_O_3_ usually form small polarons^57,58^, which are self-trapped at the localized potential wells at the Fe cations, due to the strongly polar nature of the lattice. The dynamics of phonon-driven polarons is significantly slower than that of magnetic interactions relevant for our work here. Hence, we surmise that H-dopants reduce the neighbouring Fe^3+^-cations to behave effectively like Fe^2+^-cations. We now use this understanding to build a magnetic model.

### Effect of electron injection on magnetic anisotropy

To understand the observed change of anisotropy and Morin transition, we built a phenomenological model describing their evolution as a function of temperature and H-doping. Based on the discussion in the previous section, the model consists of a matrix of Fe^3+^-cations (in magnetic sublattices ${\mathbf{M}}_{1,2}$) interspersed with sparsely distributed Fe^2+^-cations, proportional to the H-concentration. The evolution of the anisotropy in α-Fe_2_O_3_ is described primarily by the axial term in the free energy density: ${F} = {K}{{\sin} ^{2}}\theta$, where *θ* is the angle between ${\mathbf{{M}}}_{1,2}$ and the *c*-axis (see Supplementary-S11). *K* originates from two competing contributions^19,26,50^: (i) the classical magnetic-dipolar anisotropy, following a ${{K}_{\mathrm{MD}}} {\propto} {\langle{\hat S}_{z}\rangle}^{2}$ spin dependence and favouring an easy-plane orientation, and (ii) the cooperative action of crystal-field-splitting and spin–orbit-coupling Hamiltonians, termed single-ion anisotropy, following a $K_{{\mathrm{SI}}} \propto \langle\hat S_z^2\rangle$ spin dependence and supporting an easy-axis orientation. In α-Fe_2_O_3_, these interactions are opposite in sign but similar in magnitude (differing by only ~2% in bulk at 0 K), with *K*_SI_ (*K*_MD_) dominating below (above) $T_{{\mathrm{M}}_0}$, respectively, due to their slightly different temperature dependences^19,50^. Hence, the total anisotropy, $K\left( T \right) = K_{{\mathrm{MD}}}\left( T \right) + K_{{\mathrm{SI}}}(T)$, undergoes a sign reversal at $T_{{\mathrm{M}}_0}$ resulting in the Morin transition (see Fig. 1a). Essentially, the role of H-dopants is then to modify the temperature evolution of the total anisotropy by delicately tuning the relative strengths of the magnetic-dipolar and single-ion terms via the aforementioned electron injection.

Quantitatively, in our experimental range of small H-doping, *K* would evolve approximately linearly^19,26^ with the H-concentration, *x*, as per the relation (see Supplementary-S11, S12 for details):

1 $$K\left( {x,T} \right) \approx K_{{\mathrm{MD}}}\left( T \right) \left[ {1 - 2ax} \right] + K_{{\mathrm{SI}}}^{{\mathrm{Fe}}^{3 + }}(T) [1 - ax] + K_{{\mathrm{SI}}}^{{\mathrm{Fe}}^{2 + }}(T) [ax].$$

Here, the first term is the magnetic-dipolar anisotropy contributed by the spins, exhibiting the usual temperature dependence governed by the Brillouin function^50^, $K_{{\mathrm{MD}}}(T) = K_{{\mathrm{MD}}}\left( {T = 0} \right)B_{\mathrm{S}}^2\left( {Z\left( T \right)} \right)$ (see Supplementary-S11). The proportionality factor *a* simply converts the atomic H (or Fe^2+^) concentration (*x*) into the ratio of Fe^2+^-species per total Fe cations. The first term in Eq. (1) describes the weakening of the effective magnetic-dipolar interaction upon H-doping due to the reduced spin contribution from the Fe^3+^ cations. The second and third terms in Eq. (1) describe the single-ion anisotropy of the Fe^3+^- and Fe^2+^-cations, respectively, which have temperature dependences of the form^50^,

2 $$K_{{\mathrm{SI}}}^i\left( T \right) = \frac{{K_{{\mathrm{SI}}}^i\left( {T = 0} \right)}}{{\left( {2S_i - 1} \right)}}\left[ {2\left( {S_i + 1} \right) - 3B_{S_i}\left( {Z_i} \right)\coth \left( {\frac{{Z_i}}{{2S_i}}} \right)} \right],$$

where *i* represents the cations (Fe^3+^ or Fe^2+^), and *Z*_*i*_(*T*) are cation-dependent functions. The latter are obtained from a modified two-sublattice mean-field model (Supplementary-S11, S12), which describes the coupling of Fe^2+^-cations with a ‘bath’ of Fe^3+^-cations. Here, H-doping reduces the original Fe^3+^-cation single-ion interaction, accompanied by an enhancement of the Fe^2+^ term. The underlying physics is that the Fe^2+^-cations are in an effective D state with partially unquenched angular-momentum. This allows a first-order coupling which is significantly stronger^19,26^ than its counterpart in Fe^3+^-cations, which are in an S state. Further, $K_{{\mathrm{SI}}}^{{\mathrm{Fe}}^{2 + }}(T = 0)$ is opposite in sign to $K_{{\mathrm{SI}}}^{{\mathrm{Fe}}^{3 + }}(T = 0)$ and favours spins to lie in the plane (see Supplementary-S13). Hence, a few percent of Fe^2+^-cations can sizably decrease the combined single-ion contribution of the Fe cations. In our phenomenological model, the single-ion factor of the Fe^2+^-cation (labelled $D_{{\mathrm{SI}}}^{{\mathrm{Fe}}^{2 + }}$ and discussed in Supplementary-S11, S13), which depends on the splitting between ground and excited states of Fe^2+^ in H-doped α-Fe_2_O_3_ lattice, is not defined in the literature and is therefore treated as an adjustable parameter to best fit the experimental results.

Hence, the overall effect of hydrogenation is to reduce the single-ion contribution more than the magnetic dipolar counterpart, thereby suppressing the Morin transition. The temperature dependence of the total anisotropy *K*(*T*), calculated from our phenomenological model for different *x* values, is shown in Fig. 2b, while Fig. 2c shows the change in the total anisotropy at 0 K as a function of *x*, ${{\Delta }}K(x,T = 0)$. The evolution of the relative Morin transition temperature as a function of doping (i.e. $T_{\mathrm{M}}(x)/T_{{\mathrm{M}}_0}$), calculated from our model by solving the equation, $K\left( {x = {\mathrm{const}},T_{\mathrm{M}}} \right) = 0$, is plotted as the red line in Fig. 2a (lower abscissa). We observe that beyond a threshold H-concentration, $K\left( T \right) \,< \,0$ at all temperatures, leading to a complete suppression of Morin transition and the 90° switching of the Néel vector direction (for $T \,< \,T_{{\mathrm{M}}_0}$). The phenomenological curve closely resembles the experimental trend realized by tuning the H-spillover temperature, in Fig. 2a (upper abscissa).

To test the validity of our model, we also performed DFT calculations of the total anisotropy change, ${{\Delta }}K_{{\mathrm{DFT}}}$, caused due to addition of *H*_i_-defects in α-Fe_2_O_3_ (see Supplementary-S17). Calculations were performed for three hexagonal cell-sizes (2 × 2 × 1;2 × 1 × 1;1 × 1 × 1) to emulate the variation of H-concentration in the oxide. In each case, the *H*_i_ configuration was initialized from eight different doping sites for exhaustiveness (see Supplementary-S14). The evolution of ${{\Delta }}K_{{\mathrm{DFT}}}$ for each case is outlined in Supplementary-S17. The configuration giving maximum anisotropy change for each cell-size (i.e. H-concentration), ${{\Delta }}K_{{\mathrm{DFT}}}^{{\mathrm{max}}}(x)$, was then chosen for Fig. 2c. Our DFT calculations only provide the information about the ground state at *T* *=* 0 K. We find that the evolution of ${{\Delta }}K_{{\mathrm{DFT}}}^{{\mathrm{max}}}(x)$ closely follows that of ${{\Delta }}K(x,T = 0)$, obtained from our magnetic model, though the scaling factor is off by about half (see Supplementary-S17). Nonetheless, this qualitative equivalence validates the phenomenological model and confirms the anisotropy changes which govern the antiferromagnetic evolution in α-Fe_2_O_3_ under H-doping.

### Reversible control of room-temperature spin orientation

**Fig. 4:**
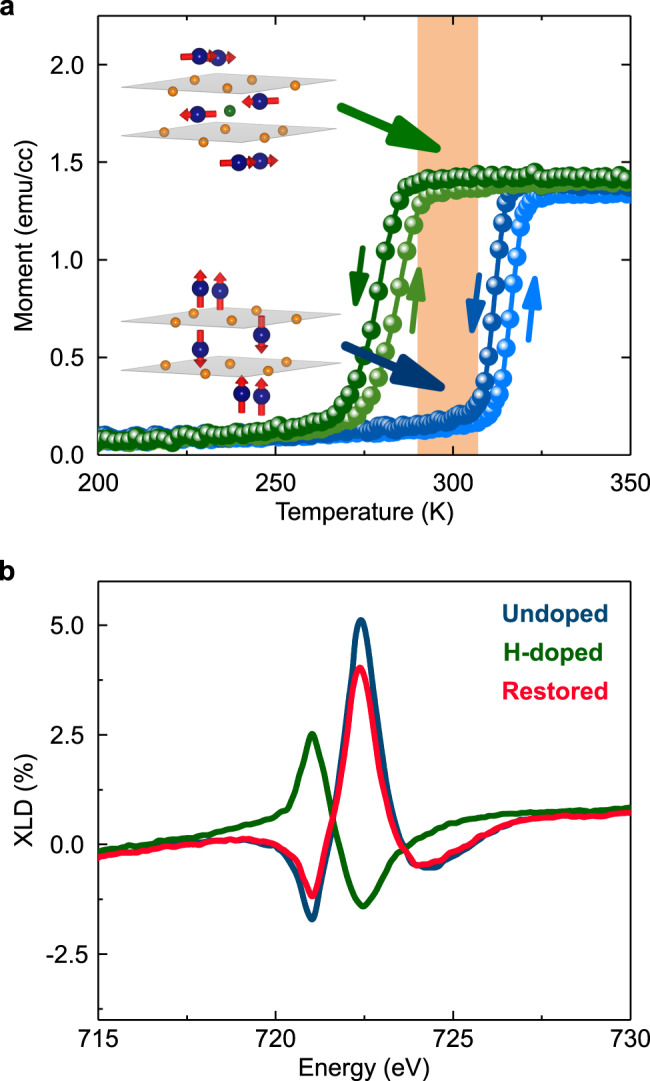
Room-temperature reversible control of the antiferromagnetic Néel vector orientation and the Morin transition. **a** *M*(*T*) curves of undoped α-Fe_1.97_Rh_0.03_O_3_ (blue) and H-doped α-Fe_1.97_Rh_0.03_O_3_ (green) samples during up/down temperature sweeps in a measuring field of 500 Oe (following field cooling at 500 Oe). The insets correspond to partial crystal unit-cells in undoped (lower) and H-doped (upper) scenarios, with 90° switched **L** orientations in the orange shaded temperature region. **b** XLD spectra at the Fe L_2_ edge of the undoped, H-doped and restored α-Fe_1.97_Rh_0.03_O_3_ samples at 286 K, exhibiting reversal of dichroism, and thereby the Néel vector orientation, as shown in the inset of (**a**). Detailed reversibility results are given in Supplementary-S4.

Lastly, we demonstrate that catalytic H-spillover can achieve reversible control of the room-temperature AFM-state as well. Key to this result is the observation that substituting a small fraction of Fe sites in α-Fe_2_O_3_ with a heavy transition metal increases its total anisotropy, thereby increasing the Morin transition temperature^19,27,30^. Here, we used α-Fe_1.97_Rh_0.03_O_3_ films which have an elevated Morin transition near room temperature (Fig. 4a and Supplementary-S2). Given that Rh^3+^-cations are isoelectronic with Fe^3+^-cations, this enhancement of *T*_M_ in the as-grown sample is not an electronic doping effect. Rather, the presence of Rh^3+^ enhances the single-ion anisotropy relative to the magnetic-dipolar term^19^, resulting in a higher *T*_M_.

Catalytic spillover of hydrogen into these Rh-substituted films, using the same annealing process as before (see Supplementary-S4), lowers the transition systematically. Moreover, in the temperature range demarcated orange in Fig. 4a, we observed a complete reorientation of **L** as identified by Fe L_2_ edge XLD, Fig. 4b. It should be noted that, unlike the case of α-Fe_2_O_3_ (Fig. 1e), we did not observe a complete suppression of the Morin transition in α-Fe_1.97_Rh_0.03_O_3_ even after hydrogenation at 250 °C, most likely due to the fact that baseline single-ion anisotropy is much higher upon Rh-substitution^19^. By all evidence, the additional in-plane anisotropy induced by H-doping counteracts the effect of Rh, weakening the overall single-ion anisotropy as outlined by our magnetic model (Fig. 2). As in α-Fe_2_O_3_, the original room temperature state of α-Fe_1.97_Rh_0.03_O_3_ was restored by annealing H-doped samples in 100% Ar or O_2_ atmospheres, as seen in Fig. 4b and Supplementary-S4. This shows that magnetic anisotropy at room temperature is controllable and reversible through H-doping, which is important for potential applications.

### Summary and outlook

In conclusion, we have demonstrated reversible control of antiferromagnetic properties up to room temperature in epitaxial thin films of insulating α-Fe_2_O_3_ and α-Fe_1.97_Rh_0.03_O_3_ by H-doping. Hydrogenation leads to electron injection, strongly influencing the delicate balance between magnetic-dipolar and single-ion anisotropy terms. Hence, a little hydrogen can dramatically change the Morin transition and thence the Néel vector direction. Although the spillover approach of ion control shown here is a diffusive process, limiting direct device implementations, it serves as a scientific proof-of-principle opening multiple avenues for future exploration. Firstly, it may inspire investigations of ion-based reversible control of antiferromagnetism in broad families of transition-metal oxides that may be able to absorb small amounts of hydrogen^59,60^, such as orthoferrites^61–63^, orthochromites^64^ and layered iron-perovskites^65^, which host first- or second-order Morin transitions^61,63–65^. Secondly, the ionic control could also be used for on-demand patterning of magnetic anisotropy. Thirdly, driving ionic motion with electric-fields, emulating state-of-art magneto-ionic proton-pumps^45,66^ that exhibit ms-timescale ionic switching^66^, may enable practical, non-volatile and reversible control over magnons^6,20,21^ or topological AFM textures^22–24^ in α-Fe_2_O_3_ and related systems. Lastly, given our observation that anisotropy modulation here originates essentially from electron-transfer, it would be interesting to control the AFM-state by ferroelectric gating at room temperature, which could operate at significantly faster timescales.

## Methods

### Film growth and H-spillover treatment

α-Fe_2_O_3_ and α-Fe_1.97_Rh_0.03_O_3_ epitaxial thin films were grown by pulsed laser deposition (PLD) from stoichiometric targets on single crystalline α-Al_2_O_3_ substrates (CrysTec GmbH), using KrF excimer laser (248 nm). Substrates were cleaned by ultra-sonication in high purity acetone, alcohol and DI Water prior to deposition. The growth was performed at 700 °C, in oxygen partial pressure of 2/20 mTorr with a laser repetition rate of 3 Hz. Subsequently, in situ high oxygen pressure annealing was performed to minimize oxygen vacancies formed during growth. The films were cooled at 10 °C/min.

Nano-sized Pt islands used for catalytic H-spillover were grown by sputtering in an Ar atmosphere from a high purity Pt target. The deposition current was 10 mA and duration was 5 s. HAADF mode in STEM identified that Pt formed small and discontinuous nano-structures (NSs) (see Supplementary-S3). Hydrogenation and various control annealing treatments were carried out in a custom-built gas flow system containing mass-flow-controllers and an internal heating stage. For hydrogenation, Pt NS-covered samples were annealed in high purity forming gas (H_2_/Ar ratio of 5%/95%) at annealing temperatures in the range 150–270 °C. Control annealing experiments were carried in high purity 100% Ar or O_2_ atmospheres under the same temperature conditions.

### Materials characterization

Structural characterization of epitaxial films included high-resolution synchrotron X-ray diffractometry (HR-XRD) involving $2\theta - \omega$, rocking curves (*ω*-scans), reciprocal space mapping (RSM) and *ϕ*-scans, and thickness measurements via X-ray reflectivity (XRR) at a four-circle diffractometer in the XDD beamline at the Singapore Synchrotron Light Source (SSLS), see Supplementary-S2, S8. Accurate lattice constants were obtained from the transformation of reciprocal space vectors (Supplementary-S8). Films for XAS dichroism and magnetometry experiments were in the thickness range of ~20–45 nm (unless specified otherwise) to ensure that they were structurally relaxed. Film thickness was measured for sample batches to allow volumetric normalization and comparison. Thicker films in the range 45–60 nm were used for XAFS and ion-beam experiments as they required higher sample interaction volume to improve the signal to noise ratios.

Atomic positions in the films and the quality of the film-substrate interface were determined through atomic-resolution STEM in a JEOL-ARM200F (with ASCOR aberration corrector and cold-field emission at 200 kV), in HAADF and annular bright field (ABF) acquisition modes. The HAADF and ABF images were acquired with a probe‐forming aperture of 30 mrad and collection inner and outer semi-angles in the range 68–280 mrad and 12–24 mrad, respectively. While HAADF shows the positions of the heavier elements Fe and Al, ABF shows the positions of O better.

For the Fourier transform infrared (FTIR) spectroscopy experiments, MIRacle ATR sampling accessory for analysis of solids was used. MIRacle was placed in the sample chamber of Vertex 80 v Bruker spectrometer. α-Fe_2_O_3_ (H-doped and undoped) fine powder samples were placed directly on the crystal for data collection. The spectra were obtained by averaging multiple curves collected using a DTGS detector, with a spectral resolution of 4 cm^−1^, scanner velocity 10 kHz and sampling time of 1 min (68 scans). The bench of the spectrometer was evacuated down to ~1.5 mbar for all experiments. The FTIR spectra were fitted by Lorentzian functions to identify the bond peaks.

### X-ray absorption fine structure (XAFS)

Evolution of Fe-valence^67^ and molecular bonding changes in α-Fe_2_O_3_, before and after H-doping, were measured by employing Fe K edge X-ray absorption near-edge structure (XANES) and extended X-ray absorption fine structure (EXAFS), respectively, at the XAFCA beamline in SSLS. The measurement was performed in fluorescence geometry and was sensitive to the Fe-signal throughout the films. Normalization, background correction, and analysis were performed in the Athena software. Following the approach in iron-oxide literature, the fraction of reduced Fe^2+^ species with respect to Fe^3+^ species in H-doped and control Ar-annealed samples relative to undoped α-Fe_2_O_3_ samples, was estimated by analyzing the chemical shifts of Fe K XANES (see Supplementary-S9).

### X-ray magnetic dichroism

Soft X-ray dichroism measurements with radiation polarised linearly (XLD) and circularly (XMCD) were carried out at the Fe L edge at BL4.0.2 of the Advanced Light Source (ALS). The measurement was performed in a transmission geometry with the detection in the luminesce-yield mode (which is a transmission measurement using sapphire as a scintillator and is thus sensitive to X-ray absorption through the entire film) in an open-flow liquid nitrogen cryostat which allowed dichroism measurements in the range 80–325 K. X-rays were incident with a grazing angle of 30° relative to the surface. For the XLD asymmetry, defined as $(I_{{\mathrm{LVP}}} - I_{{\mathrm{LHP}}})/(I_{{\mathrm{LVP}}} + I_{{\mathrm{LHP}}})$, the linear horizontal polarization (LHP) was parallel to basal planes. X-ray incidence was kept fixed with respect to in-plane notch direction for all samples, to eliminate any structural dichroism variation between samples. We can observe the spin reorientation across the Morin transition directly through the XLD experiment, as discussed above. A 90° change in the antiferromagnetic spin-orientation of α-Fe_2_O_3_ across the Morin transition causes a sign reversal of the dichroic signal at the Fe L_2a_ and L_2b_ edges. This is consistent with the dichroic reversal previously observed^51^ in α-Fe_2_O_3_ literature across the Morin transition. It should be noted that in α-Fe_2_O_3_ literature^25,51^, the XLD is attributed to magnetic and not structural contributions (see Supplementary-S6 for details).

### Temperature- and field-dependent magnetometry

Magnetic characterization was performed in a superconducting quantum interference device (SQUID) magnetometer of Quantum Design MPMS. *M*(*T*) curves were measured after field-cooling the samples with an applied in-plane field of 500 or 5000 Oe, and a measurement field of 500 Oe applied during the subsequent warming and cooling scans. Changing the cooling-field magnitude had no discernible effect on the transition temperature. *M*(*H*) curves were carried out at a series of temperatures. The diamagnetic α-Al_2_O_3_ substrates contribute a background in the raw *M*(*H, T*) data. This background is removed to extract the final magnetometry curves of the thin films.

### Ion-beam analysis techniques (RRBS, ERDA)

The quantitative analysis of H-dopant concentration in samples was performed using elastic recoil detection analysis (ERDA) wherein He ions incident on the sample lead to selective forward recoil scattering of H atoms from the sample. ERDA experiments were performed in tandem with oxygen resonant Rutherford backscattering spectrometry (RRBS) at Inter-University Accelerator Centre (IUAC), using 1.7 MV pelletron Tandem accelerator. The ERDA and RRBS measurements facilitated a direct quantitative determination of Fe/O/H concentrations simultaneously in the samples. RRBS experiments were performed with a resonant energy for oxygen reaction ^16^O(α, α)^16^O, to accurately measure the O-content in the α-Fe_2_O_3_ films (see Supplementary-S7). Thin films were mounted on a 4-axis goniometer and the chamber was maintained at a pressure ~10^−6^ Torr. The He ions were incident at an angle of 75°. Backscattered He (for RRBS) and recoiled H (for ERDA) were collected in surface-barrier detectors placed at 165° and 30° with respect to the incident ions, respectively. A 14-μm-thick Al absorber-foil was used to stop the forward-scattered He and only allow recoiled H toward the ERDA detector. Both ERDA and RRBS spectra were fit using the SIMNRA software. For RRBS experiments, the energy calibration was performed by using a standard tungsten silicide sample. To calibrate the yield and energy of ERDA measurements, a quantitative analysis of H density was obtained by using an H-implanted Si standard and by performing ERDA measurements at two energies in the range ~2.80-3.04 MeV.

### First-principles calculations

Density functional theory (DFT) based first-principles calculations were performed through the Vienna Ab-initio simulation package (VASP). The projector-augmented-wave and the Perdew–Burke–Ernzerhof (PBE) method were used for the pseudo-potentials and the exchange-correlation functionals, respectively. The plane-wave cut-off energy was set at 600 eV. The GGA+U approach^68^ was used for the *d*-electrons of Fe atoms, and the value of U was set to 4.5 eV, which brings the indirect electronic bandgap of *α*-Fe_2_O_3_ from 0.56 eV (PBE result) to 2.0 eV, consistent with our experimental bandgap value from UV-Vis-NIR absorption spectroscopy. In addition, spin–orbit coupling (SOC) was also involved in all the calculations to accurately obtain information related to magnetic anisotropy.

For hydrogen-doping calculations, one H atom was incorporated into 1 × 1 × 1, 2 × 1 × 1, and 2 × 2 × 1 unit-/super-cells (hexagonal) of *α*-Fe_2_O_3_, respectively, to mimic different doping concentrations. The Monkhorst–Pack grid with 7 × 7 × 3, 7 × 3 × 3, and 3 × 3 × 3, k-point meshes were used, respectively. For each of these cases, defects both of the interstitial (*H*_i_) and substitutional (*H*_O_) type were considered. In addition, eight different initial sites for *H*_i_ were considered, shown in Supplementary-S14. We performed DFT calculations allowing atomic Fe/O/H positions to evolve and relax until the Feynman–Hellman forces between atoms became smaller than 0.01 eV/Å, while keeping lattice constants fixed. For consistency, change of lattice constants was ignored in the phenomenological model in Supplementary-S11–S13. The defect formation energies were calculated from the relation,

3 $$E_f\left( X \right) = E_{{\mathrm{tot}}}\left( X \right) - E_{{\mathrm{tot}}}\left( {{\mathrm{bulk}}} \right) - \mathop {\sum}\nolimits_j {n_j\mu _j} ,$$

where *E*_tot_(*X*) and *E*_tot_(bulk) are the calculated total energies of the system with and without defect *X*, respectively. *n*_*j*_ is the number of atoms of type *j* (either host atoms or dopants) that have been added to (*n*_*j*_ > 0) or removed from (*n*_*j*_ < 0) the system to form that defect, and *μ*_*j*_ is the corresponding chemical potential of the species. Here, the chemical potential of H and O are obtained by considering hydrogen and oxygen molecules as their sources, respectively.

Moreover, defects/host atoms can further acquire or lose electrons, forming charged states. This effect is simulated by performing the calculation with excess electron(s) added to or removed from the cell, respectively. The relative stability of each charged state of the cell containing the defect can be measured by modifying the above formation energy in Eq. (3) as follows^69^,

4 $$E_f\left( X \right) = E_{{\mathrm{tot}}}\left( X \right) - E_{{\mathrm{tot}}}\left( {{\mathrm{bulk}}} \right) - \mathop {\sum}\nolimits_j {n_j\mu _j + q\left( {E_{\mathrm{F}} + E_{{\mathrm{VBM}}} + {{\Delta }}V} \right)}$$

where the *q* indicates the charged state, *E*_F_ is the Fermi energy, *E*_VBM_ the reference valence band maximum (VBM) position, and Δ*V* is a correction term added to align the VBM level of the cell containing the defect (in different charge states) to that in undoped *α*-Fe_2_O_3_, as discussed in ref. ^69^.

Lastly, to analyse charge transfer between atoms in H-doped *α*-Fe_2_O_3_, we adopted the Bader-charge calculations as it considers not only the core charge but also the valence charge, serving as a good approximation of the total electronic charge.

## Supplementary information

Supplementary Information

## Data Availability

The data that support the findings of this study are available within the article and its Supplementary Information file. Materials are available from the corresponding authors upon reasonable request.
