# Supplementary material for: Reversible hydrogen control of antiferromagnetic anisotropy in α-Fe2O3
Source: Nat Commun. 2021 Mar 12;12:1668. doi: 10.1038/s41467-021-21807-y (PMC7954816; doi:10.1038/s41467-021-21807-y)
Supplement: Supplementary file 1 — Supplementary Information [file 41467_2021_21807_MOESM1_ESM.pdf]

Supplementary Information for

Reversible hydrogen control of antiferromagnetic anisotropy in α-Fe_2O_3

Hariom Jani*, Jiajun Linghu, Sonu Hooda, Rajesh V. Chopdekar, Changjian Li, Ganesh Ji Omar, Saurav Prakash, Yonghua Du, Ping Yang, Agnieszka Banas, Krzysztof Banas, Siddhartha Ghosh, Sunil Ojha, G. R. Umapathy, Dinakar Kanjilal, A. Ariando, Stephen J. Pennycook, Elke Arenholz, Paolo G. Radaelli, J. M. D. Coey, Yuan Ping Feng, T. Venkatesan*

*Correspondence should be sent to hariom.k.jani@u.nus.edu or venky@nus.edu.sg

Contents:

1. Supplementary Sections – 17
2. Supplementary Tables – 3
3. References

S1. Magnetic anisotropy and crystal orientation

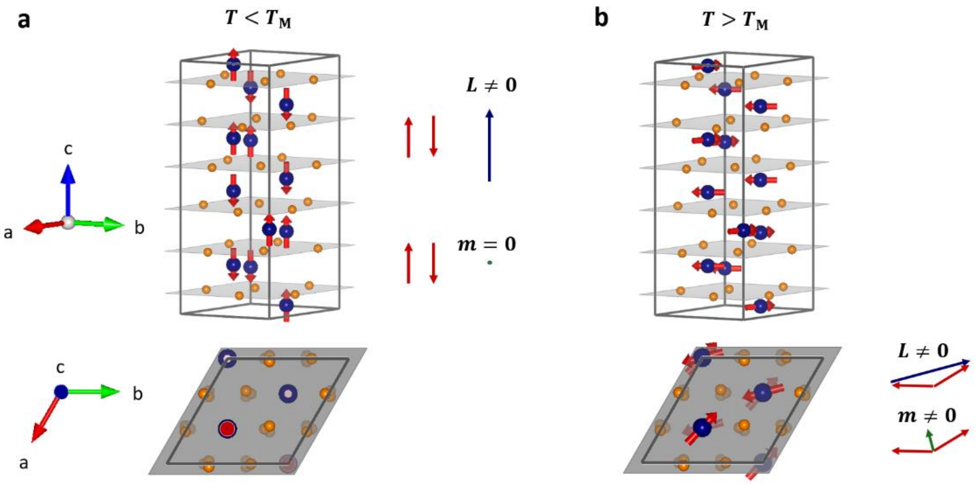

Fig. S1.1 | Schematic representation of the α-Fe_2O_3 unit-cell along different axes, below (**a**) and above (**b**) the Morin transition. Fe (O) atoms are blue (orange) balls, and sublattice magnetizations are red arrows (not to scale). The crystal axes are also indicated to the left. The Néel-vector – $\boldsymbol{L}$, and the net canted moment – $\boldsymbol{m}$, are indicated as thin blue and green arrows, respectively. $\boldsymbol{L}$ lies out-of-plane (in-plane) below (above) the Morin transition[1]. Canting angles in (b) are exaggerated for clarity.

S2. Structural quality of epitaxial films

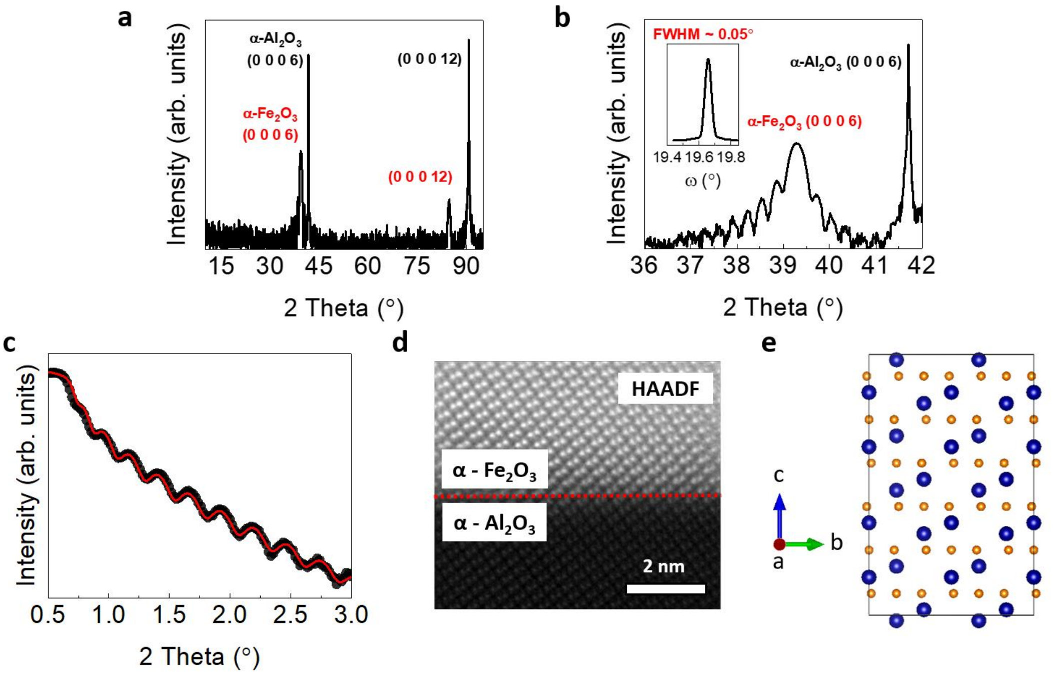

Fig. S2.1 | Structural quality of α-Fe_2O_3 films. (**a**) Wide-angle XRD and (**b**) high-resolution (HR)-XRD $2\theta-\omega$ scans with the film and substrate peak positions indicated. The rocking curve of the film in (b) is given in inset. (**c**) XRR scan of a film (black dots) and fit (red line) indicating a thickness of ~30 nm. (**d**) A HAADF-STEM image of the film-substrate interface. The brighter spots are Fe atoms in the film and lighter spots correspond to Al atoms in the substrate. (**e**) Schematic of the corundum cell structure showing the Fe positions (blue) relative to O atoms (orange) and the viewing axis.

α-Fe_2O_3 films were grown epitaxially on c-cut α-Al_2O_3 substrates by PLD (see 'Methods'). Films exhibit good coherence and smoothness, attested by Laue fringes in HR-XRD and Kiessig fringes in XRR, respectively (**Figs. S2.1b**,**c**). Films also possessed sharp rocking curves with narrow full-width-half-maximum, evincing their high epitaxial quality (**Fig. S2.1b** inset). Scanning tunnelling electron microscopy (STEM-HAADF mode) revealed a sharp interface and good coherence (**Fig. S2.1d**).

Similarly, high quality epitaxial thin films of α-$Fe_{1.97}Rh_{0.03}O_3$ were also grown on α-Al_2O_3 substrates (**Fig. S2.2a**), at the same growth conditions. The magnetism and anisotropy properties of α-$Fe_{1.97}Rh_{0.03}O_3$ films studied via magnetometry and XLD revealed the anisotropy reversal across the Morin transition (**Fig. S2.2b**) which is now elevated relative to non-Rh substituted samples.

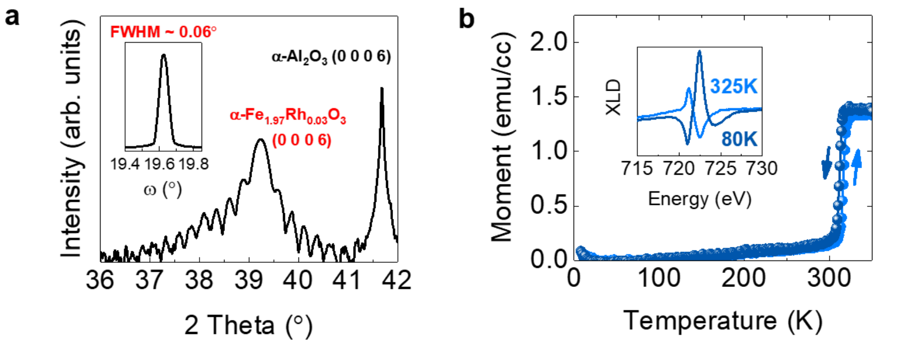

Fig. S2.2 | Properties of α-$Fe_{1.97}Rh_{0.03}O_3$ films. (**a**) HR-XRD $2\theta-\omega$ scans with the film and substrate peak positions indicated. The rocking curve of the film peak is given in the inset. (**b**) Temperature dependent magnetometry curve (warming-cooling). Inset shows the XLD contrast above (below) the Morin transition, where the $\boldsymbol{L}$ lies in-plane (out-of-plane).

S3. Platinum nanostructures for catalytic H-spillover

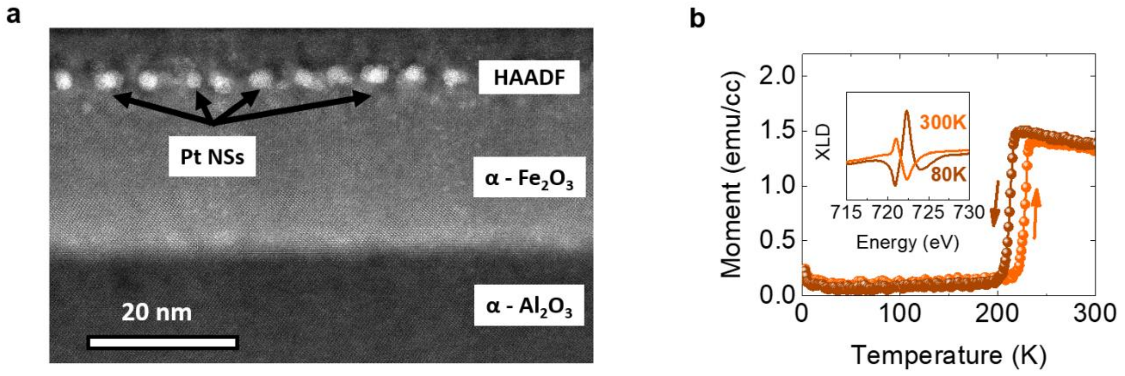

Fig. S3.1 | Undoped α-Fe_2O_3 film decorated with Pt nano-structures. (**a**) STEM-HAADF image of discontinuous sputter grown Pt on top sample surface. (**b**) Magnetometry curve obtained during up and down temperature sweep. Inset shows the XLD above and below the Morin transition.

Discontinuous platinum (Pt) grown after sputter deposition, formed spheroidal nano-structures (NSs) of diameter slightly more than ~2 nm on the top surface of the sample (**Fig. S3.1a**). Due to Z-contrast of the HAADF mode, Pt appears brightest. The ratio of the Pt areal density obtained from the RBS measurements to the theoretical value expected from a continuous Pt layer was much less than unity, confirming partial surface coverage of Pt nano-structures. Magnetometry of Pt NS-covered α-Fe_2O_3 film (**Fig. S3.1b**) showed the comparable $M(T)$ and X-ray dichroism as the undoped α-Fe_2O_3

film without Pt nanostructures, confirming that the Pt plays a catalytic role only, and that it does not contribute any changes to the magnetic properties of α-Fe_2O_3.

S4. Catalytic Spillover of Hydrogen

Annealing temperature control

Catalytic H-spillover was performed as described in the ‘Methods’ section. **Fig. S4.1a** demonstrates that a low-temperature annealing step (≤ 270 °C), activates bulk diffusion of hydrogen into α-Fe_2O_3, with no significant structural changes in the crystal oxide lattice. However, treating α-Fe_2O_3 at higher temperatures (≥ 300 °C), **Fig. S4.1b**, caused the nucleation of Fe_3O_4. SQUID measurements on latter samples (not shown here) revealed a magnetization 2 orders of magnitude larger along with a clear Verwey transition at T_V ~ 120 K, as expected in the Fe_3O_4 phase. Hence, thermally driven H-spillover has an upper-limit on the amount of H incorporation than can be achieved without the formation of O-vacancies or Fe_3O_4 phase. As our goal was to investigate the H-doped α-Fe_2O_3 phase and not Fe_3O_4, we performed all hydrogen annealing experiments strictly below 270 °C.

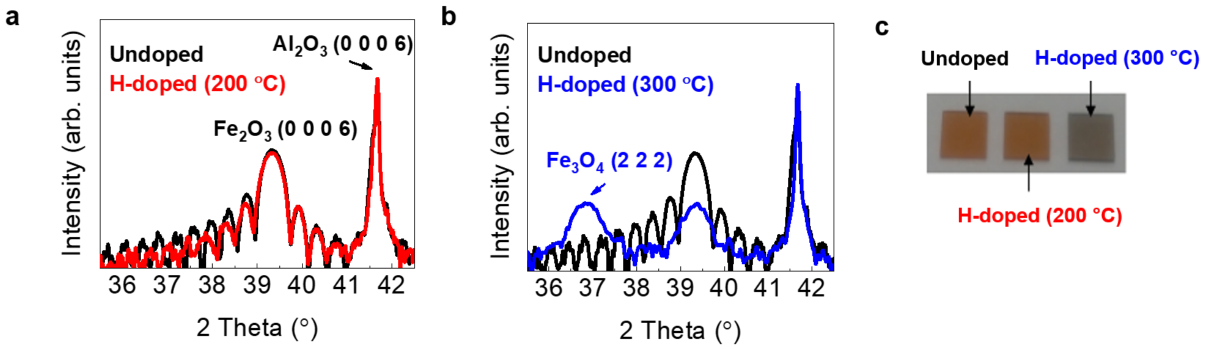

Fig. S4.1 | Annealing temperature control. (**a**) $2\theta-\omega$ scans of the α-Fe_2O_3 sample H-doped at 200 °C showing no change in Bragg position compared to its undoped state, and very minor variations in the Laue fringes. However, catalytically H-doping the film at or above 300 °C (**b**) caused significant nucleation of Fe_3O_4 and a complete deterioration of the α-Fe_2O_3 phase. (**c**) Variation of sample colour with hydrogen annealing performed at different temperatures, confirmed the phase assignment in (b).

Annealing time control

Annealing time plays the role of allowing the catalytically inserted H-dopants to diffuse homogenously across the sample. We found that much shorter duration of H-spillover (2, 5, 15 min) for optimal doping conditions is also sufficient to drive the anisotropy changes (see **Figs. S4.2a-c**) just as those seen after 60 min spillover treatment (**Fig. 1d-f**). This result corresponds well with H-spillover experiments reported in other oxides (see main text H-spillover references).

Stability of H-doped state

The H-doped α-Fe_2O_3 films, prepared by annealing the samples at 250 °C, were stable at ambient temperature and pressure for 385 days (without showing any sign of degradation), **Fig. S4.2d**. This attests that the H incorporation in α-Fe_2O_3 is indeed quite robust and air-stable. We believe that this is due to the bonds that the H-dopants form with O (see Supplementary-S8 and S14).

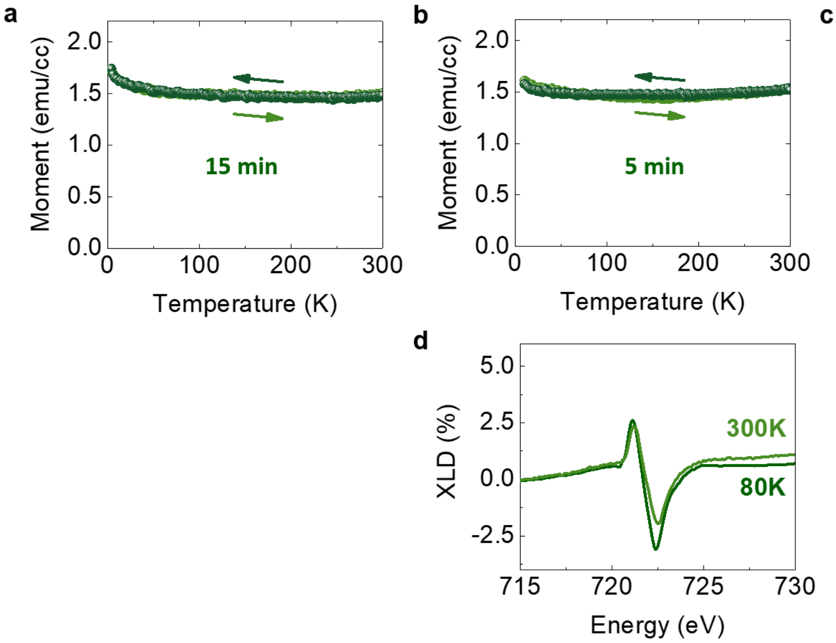

Fig. S4.2 | Annealing time control and stability. (**a-c**) Temperature dependent magnetometry curves of H-doped α-Fe_2O_3 sample prepared by annealing at 250 °C for 15 min (a), 5 min (b), 2 min (c). (**d**) Stability of the AFM state in the H-doped α-Fe_2O_3 sample (shown in main **Fig. 1d-f**), confirmed by repeated XLD measurement after 385 days.

Reversibility

After an H-doping step, the AFM-state can be restored as observed in the α-Fe_2O_3 (see **Figs. 1g-i**) as well as in α-$Fe_{1.97}Rh_{0.03}O_3$ (**Figs. S4.3a,b**) samples. The restoration process resulted in a slightly lowered T_M in comparison to the original value (T_{M_0}), such that their ratio settles to a steady value after multiple cycles (it should be noted that each step here consists of both H-insertion and removal, see inset in **Fig. S4.3b**).

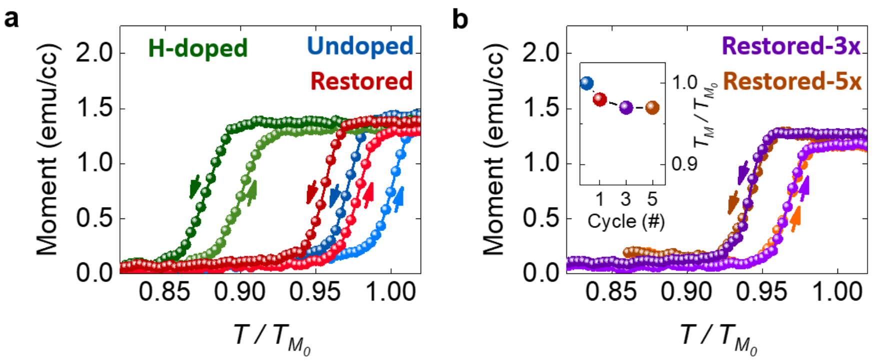

Fig. S4.3 | Restoration of the H-doped α-$Fe_{1.97}Rh_{0.03}O_3$ films. (**a**) Temperature dependent magnetometry curves of 1-time restored sample compared against the undoped and H-doped counterparts. (**b**) Equivalent magnetometry curves of 3- and 5-times restored samples. Inset shows the evolution of T_M / T_{M_0} after repeated cycling. The line is a guide to the eye.

S5. Control annealing experiments

Argon annealing of α-Fe_2O_3 films

Ar-annealing experiments, where Pt NS-covered undoped α-Fe_2O_3 films were exposed to 100% Ar for the same annealing conditions continued to exhibit the Morin transition, **Fig. S5.1**, in sharp contrast to the behaviour of H-doped films (**Fig. 1e**,**f**), implying that merely having an oxygen-absent atmosphere (as in 100% Ar) at elevated temperatures is insufficient to mimic H-spillover effects.

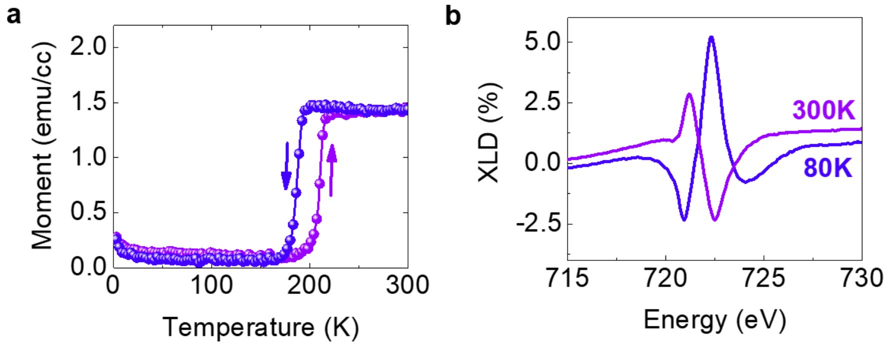

Fig. S5.1 | Magnetometry of Ar-Annealed α-Fe_2O_3 films. (**a**) Magnetometry measurements of 100% Ar-annealed α-Fe_2O_3 film. (**b**) XLD data taken at 300 K (80 K) which lies above (below) T_M , where the $\boldsymbol{L}$ lies in-plane (out-of-plane).

Absence of magnetic contribution before and after H-annealing of substrates (α-Al_2O_3)

To rule out the presence of spurious magnetic artefacts of the α-Al_2O_3 substrates (without any α-Fe_2O_3 films), we performed magnetometry of bare and H-annealed substrates. For accuracy, these control substrates were taken through the entire growth process involved in PLD film growth (heating, annealing, etc.), with one major exception that no α-Fe_2O_3 film was deposited on them. We found that α-Al_2O_3 trends are essentially diamagnetic where the $M(T)$ and $M(H)$ curves did not show any magnetic transition features (unlike α-Fe_2O_3), as Al^{3+}-cations have no net moment due to their fully-filled valence shell. H-doping also does not affect α-Al_2O_3 as the Al^{3+}-cations cannot accommodate excess electrons. These results, over and above the element specific X-ray dichroic experiments (see Supplementary-S6), clearly establish that our magnetometry data originate from α-Fe_2O_3 and its H-doped state and not the substrates or the Pt catalyst layer.

Negligible role of O-vacancies in our H-spillover experiments

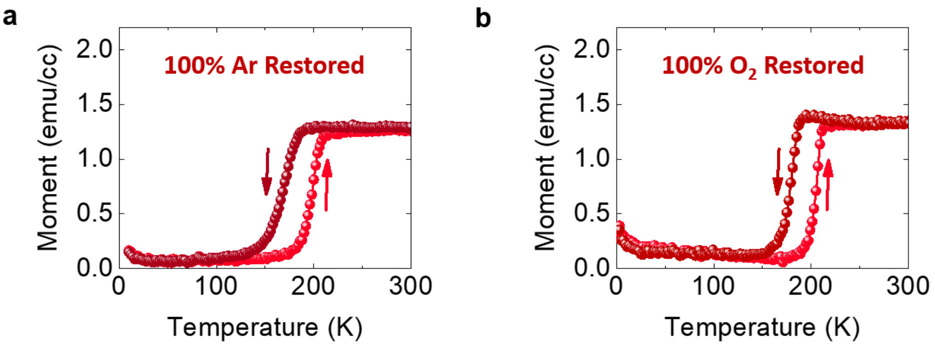

Fig. S5.2 | Magnetometry curves of restored H-doped α-Fe_2O_3 films in 100% (**a**) Ar and (**b**) O_2 atmospheres.

To discern whether the observed modulation of magnetic properties is because of H incorporation or O-vacancy creation during our H-spillover treatment (see Supplementary-S4), we performed the restoration of the H-doped samples in 100% Ar atmosphere, **Fig. S5.2a**. The Morin transition could be retrieved to a similar condition as the sample restored in 100% O_2 atmosphere, **Fig. S5.2b**, suggesting that the H-annealing step essentially adds H-dopants to the system, creating only negligible fraction of O-vacancies, as the latter cannot be healed in an O-poor atmosphere (in this case 100% Ar) at higher temperature. Here, the H-dopants certainly can undergo excorporation from the oxide in H-poor atmospheres as they are weakly bound dopants. This is consistent with other oxide H-doping studies in the literature[2-4]. We also remark that it is possible to suppress the Morin transition by growing α-Fe_2O_3 samples at 700 °C in an oxygen-poor atmosphere. However, these oxygen-deficient samples have markedly different magnetic properties from our H-doped α-Fe_2O_3 samples (not shown here). Both these results unequivocally establish that H-doping plays a distinctive role in modifying the magnetism in α-Fe_2O_3 which cannot be reproduced by merely creating O-vacancies.

S6. Fe L edge X-ray linear and circular dichroism

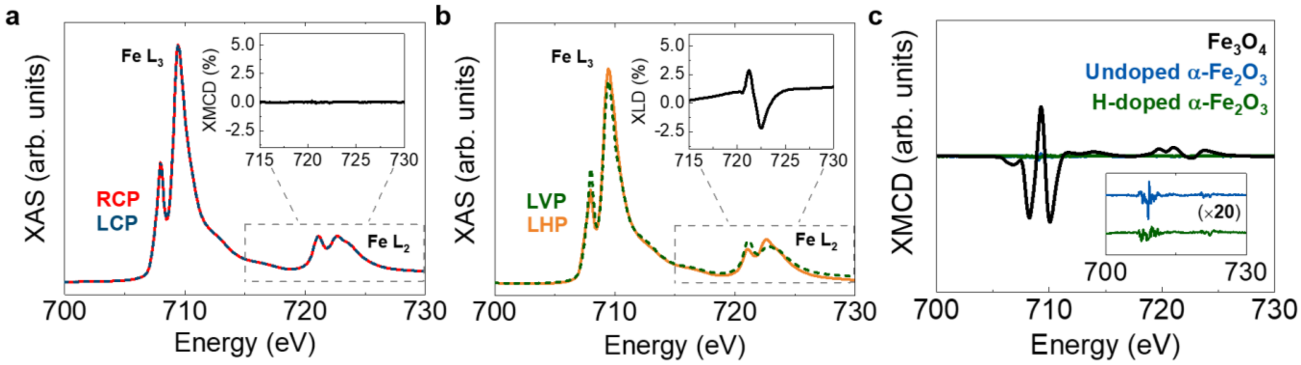

Fig. S6.1 | X-ray dichroism in α-Fe_2O_3. (**a**) XMCD and (**b**) XLD signals of α-Fe_2O_3 sample, with X-ray in grazing incidence at room temperature. RCP, LCP, LHP, LVP correspond to right-circular, left-circular, linear-horizontal, linear-vertical polarizations, respectively. (**c**) XMCD of undoped and H-doped α-Fe_2O_3 films, compared against the XMCD signal[5] of Fe_3O_4. Inset shows magnified XMCD spectra of undoped and H-doped α-Fe_2O_3 films that are translated vertically for clarity. These α-Fe_2O_3 signals lack any systematic ferromagnetic spectral features, as is well known in literature[6].

In α-Fe_2O_3, XMCD is negligible[6] whereas XLD is evident (**Figs. S6.1a,b**). According to the α-Fe_2O_3 literature, XLD has a magnetic origin here (and negligible structural or orbital contributions), as it reverses sign across the Morin transition and vanishes above the Néel temperature[7,8]. Hence, the magnetic anisotropy and Néel vector orientation (*i.e.* in-plane vs out-of-plane direction) can be readily determined from the Fe L edge XLD. Moreover, XMCD signals were absent in both undoped and H-doped α-Fe_2O_3 (**Fig. S6.1c**), due to the smallness of the canted moment[6]. This negligible XMCD signal also confirms the purity of our iron oxide samples and strongly suggests that the presence of any ferro/ferrimagnetic iron-oxide inclusions (for which XMCD is a strong tell-tale signal[9]) should be negligible in the H-doped samples prepared at optimal H-spillover temperatures used in this work (also see Supplementary-S4,S10).

S7. Selective ion beam techniques: Proof of H incorporation

Elemental analysis to study hydrogen and oxygen stoichiometry in the oxide was performed by elastic recoil detection analysis (ERDA) and oxygen resonant Rutherford backscattering (RRBS), respectively, performed in tandem (see 'Methods').

O-Detection: oxygen resonant RBS

RRBS with He-ions incident at oxygen resonance dramatically increases the back-scattering cross-section of He-ions from the surface oxygen atoms (to depths corresponding well with film thickness). Hence, He ions back-scattered from α-Fe_2O_3 contributed to the sharp O resonance peak, whereas He ions back-scattered from α-Al_2O_3 appeared as the usual oxygen hump, see **Fig. S7.1**. A quantitative summary of the O/Fe ratio for various samples is given in **Table S7.1**. These results confirm negligible difference in oxygen content between undoped, H-doped and Ar-annealed samples. This is consistent with the higher formation energies of O-vacancies as compared to H-interstitials obtained by our first principles calculations, which are discussed in Supplementary-S15.

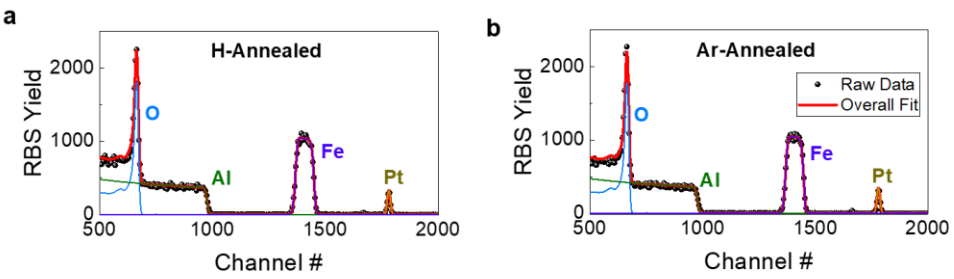

Fig. S7.1 | RRBS measurements of α-Fe_2O_3 samples (**a**) H-annealed or (**b**) Ar-annealed at 250 °C. All data were fit using SIMNRA software[10], with individual elements (O, Al, Fe, Pt) shown in separate colours.

H-Detection: ERDA

By contrast, differences in hydrogen content can be easily detected by ERDA (see **Fig. S7.2**), where the outgoing He ions and the recoiling H leave the sample in a grazing geometry. To distinguish these ion species, we placed an aluminium (Al) absorber-foil following to the standard protocol, thereby allowing only H to pass to the detector. While the foil provides selectivity, it also increases the energy straggling[11]. This increases the full-width at half-maximum (FWHM) of the surface peaks in ERDA[12], causing the signal from the surface hydrogen (either surface water[13] or hydrocarbon layer) to be as prominent as the bulk H inside the films. We subtracted the pure surface contribution (using the control undoped sample as the reference, **Fig. S7.2a**) from the total ERDA signals to extract the effective H-concentration in the bulk (for H-doped sample see **Fig. S7.2b**). The H areal density (in atoms/cm^2) was then obtained by fitting the experimental ERDA curves followed by an integration over the energy axis. A quantitative summary of the effective bulk H-concentration for all samples is given in **Table S7.1**. We observed that while the undoped and Ar-annealed samples have a small hump (coming essentially from surface adsorbed H), the H-annealed samples had a significantly higher signal, indicating the incorporation of hydrogen into the films, which is consistent with our Fourier transform infrared spectroscopy (FTIR experiments, Supplementary-S8).

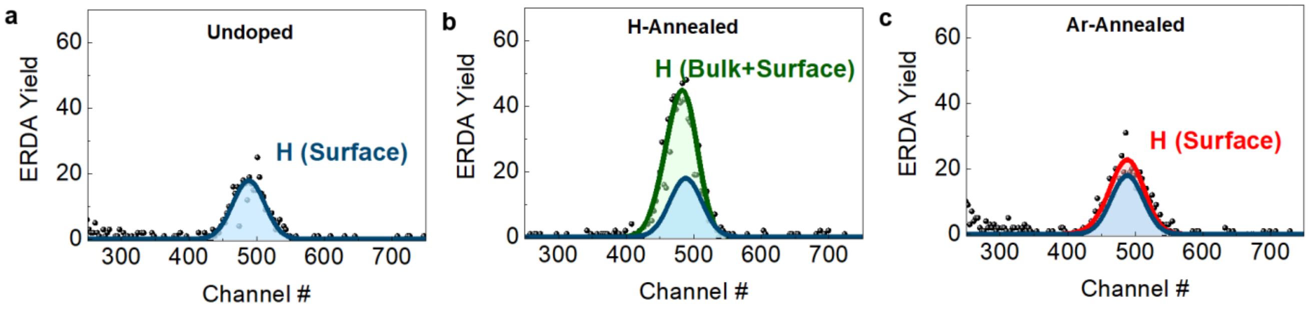

Fig. S7.2 | ERDA measurements of α-Fe_2O_3 samples that are undoped (**a**), and either H-annealed (**b**) or Ar-annealed (**c**) at 250 °C. Undoped sample had a small surface H-signal (blue) which is also benchmarked in other figures. H-doped sample (b) had a significantly higher ERDA signal (green). Ar-annealed sample (c) had effectively only a surface hydrogen signal (red).

From the quantitative results in **Table S7.1** we conclude:

- H-annealing led to incorporation of H-dopants into the α-Fe_2O_3 films with Pt NSs, by a fraction that was an order of magnitude greater than H incorporation in films without Pt NSs on the surface. This confirms that catalytic H-spillover is indeed at play in our experiments.
- Comparing samples H-annealed at 150 °C, 170 °C, 250 °C reveals that H-annealing at higher temperatures allowed greater H incorporation in the lattice. This thermodynamic activation of H incorporation follows previous reports in spillover studies[14].
- Moreover, control undoped samples with Pt nanostructures on the surface (without performing H-spillover) or those that are Ar-annealed had negligible amount of natural hydrogen adsorption.

Table S7.1 | Summary of selective He ion-beam experiments. H-concentration (at%) and O/Fe ratio for various H-annealed and control samples of similar thickness estimated based on ERDA and RRBS, respectively. H-concentration was obtained from the ratio of H areal density relative to the total counterpart for the corresponding film. The alphabet in the parentheses in the first column represents its position in **Fig. S7.2**. The error bars are from the variability in the fitting.

Sample	H-concentration (at%)	O/Fe ratio
Undoped<br>(without Pt NSs) – (a)	Used as surface hydrogen reference	1.5 ± 0.1
Undoped<br>(with Pt NSs)	0.22 ± 0.14	1.5 ± 0.1
**Hyd – Annealed – 250 °C**<br>(with Pt NSs) – (b)	**2.47 ± 0.14**	**1.5 ± 0.1**
**Hyd – Annealed – 170 °C**<br>(with Pt NSs)	**1.77 ± 0.14**	**1.5 ± 0.1**
**Hyd – Annealed – 150 °C**<br>(with Pt NSs)	**1.57 ± 0.14**	**1.5 ± 0.1**
Hyd – Annealed – 250 °C<br>(without Pt NSs)	0.25 ± 0.14	1.5 ± 0.1
Ar – Annealed<br>(with Pt NSs) – (c)	0.47 ± 0.14	1.5 ± 0.1

S8. Structural evolution with H-doping

Precise lattice constants were obtained by measurement of reciprocal space vectors (RSVs) via HR-XRD of α-Fe_2O_3 referenced against the substrate. Transformation from RSVs to real space vectors

was performed (following Ref [15]) to obtain lattice constants in **Table S8.1**. After optimal H-doping (*i.e.* in films with T_M completely suppressed) the lattice underwent mild expansion (0.23 vol%) , which is equivalent to an expansion of ~1 Å³/ H-dopant. In particular, the in-plane lattice constant expanded by ~ 0.1 %. This value is much smaller than the net expansion of ~ 0.3-0.45 % required to completely suppress T_M , purely based on a tensile strain effect when electronic doping is absent (see Ref [8]). This shows that while the effect of global strain cannot be ruled out completely in our hydrogenation experiments, it only plays a minor role at best.

Table S8.1 | Lattice constants of the hexagonal cell of the undoped and H-doped films and corresponding errors obtained from RSV measurements. The cell schematics are given in **Fig. S1.1**.

**Sample**	**a, b (Å)**	**c (Å)**	**Volume (Å³)**
Undoped	5.042 ± 0.003	13.755 ± 0.004	302.8 ± 0.3
**H-doped**	**5.047 ± 0.002**	**13.759 ± 0.004**	**303.5 ± 0.2**

Reciprocal space mapping and Phi-scans

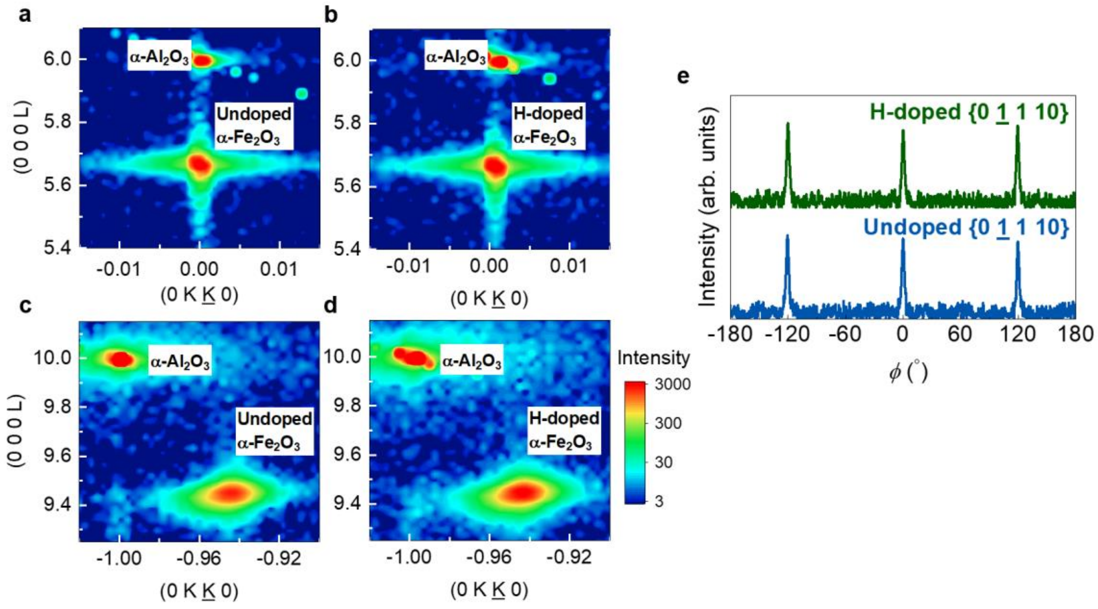

Fig. S8.1 | RSMs of undoped and H-doped α-Fe_2O_3 films around the Bragg peaks (**a**,**b**) (0 0 0 6) with purely out-of-plane component and (**c**,**d**) (0 1 1 10) with an admixture of out-of-plane and in-plane components. In-plane relaxation of the film was evident in both cases (owing to the lattice mis-match with the substrate). Upon hydrogenation, the RSMs remained mostly unchanged and no new peaks appeared in the neighbouring region. The film peaks were referenced relative to those of the substrate. (**e**) Phi-scans of the three Bragg peaks separated by in-plane rotation of 120° showed that epitaxial quality does not change before and after hydrogenation.

Scanning transmission electron microscopy

STEM measurements were performed in high-angle annular dark field (HAADF) and annular bright field (ABF) modes to study the atomic structure of H-doped α-Fe_2O_3 film on the substrate, as shown in **Fig. S8.2**. The brightest element in the upper part of the HAADF image **Fig. S8.2a** is Fe, while the lower region corresponds to Al in the substrate. We also performed ABF, where distinctive

zig-zag of atoms Fe-O-Fe and Al-O-Al (red lines) can be seen very clearly in **Fig. S8.2b**. These STEM images re-iterate that H-doping does not alter the global structure of the films.

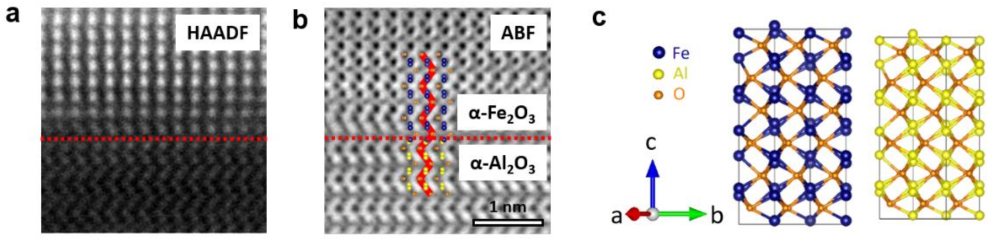

Fig. S8.2 | STEM measurements of H-doped α-Fe_2O_3 film in (**a**) HAADF and (**b**) ABF modes. Red-line shows the interface between the film and substrate. (**c**) Schematic of α-Fe_2O_3 and α-Al_2O_3 cell structures and the viewing axis for the crystal orientation in (a,b). Corresponding positions in STEM image are overlaid in the ABF image in (b).

Formation of OH bonds: FTIR

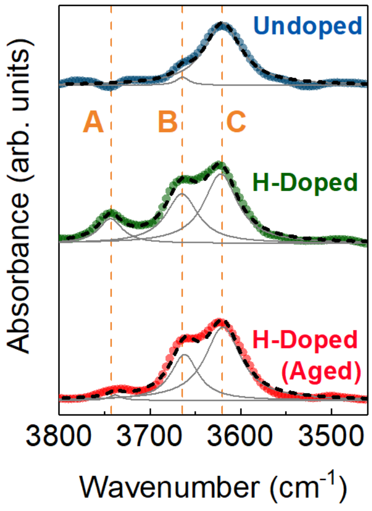

Fig. S8.3 | FTIR of undoped and H-doped α-Fe_2O_3 samples. Peak positions A, B, C are shown with dashed orange lines. The spectra were fitted with Lorentzian functions (grey). The combined fitting function for each case is shown as a dashed black curve. Spectra are baseline corrected and offset for clarity.

FTIR experiments (see 'Methods') performed in an evacuated chamber helped to identify hydrogen related bonds in H-doped α-Fe_2O_3, **Fig. S8.3**. Firstly, the lower wavenumber peak C, which was present in undoped as well as H-doped samples, may be attributed to OH in monomeric water molecules or bonds at the surface. Peak B (~3664 cm^{-1}), which was strong in the H-doped samples but also manifested as a shoulder in undoped sample, may arise likely from surface OH groups[16,17] than H-doping. Lastly, we saw a singular peak A (~3740 cm^{-1}), which uniquely manifested after H-doping and was absent in the undoped sample. We postulate this may be a signature of the new OH bonds formed after H-doping in α-Fe_2O_3. Such singular peaks related to internal OH bonds have also been reported in other hydrogenated oxide systems in the literature[18,19]. We note that this is consistent with our DFT calculations of H-dopants optimised in α-Fe_2O_3 super-cells, which revealed that incorporated H form interstitial dopants in the system and migrate to the nearby oxygen to form OH bonds (Supplementary-S14). To further verify our experimental results, we investigated another H-doped α-Fe_2O_3 sample, which was month aged. We found that all the peak positions were comparable to those in the freshly H-

doped counterpart, with peaks B, A slightly weakened in intensity presumably due to aging, as also observed previously in H-doping literature[18]. This result seems to substantiate our peak assignment.

S9. Fe K edge X-ray absorption spectra

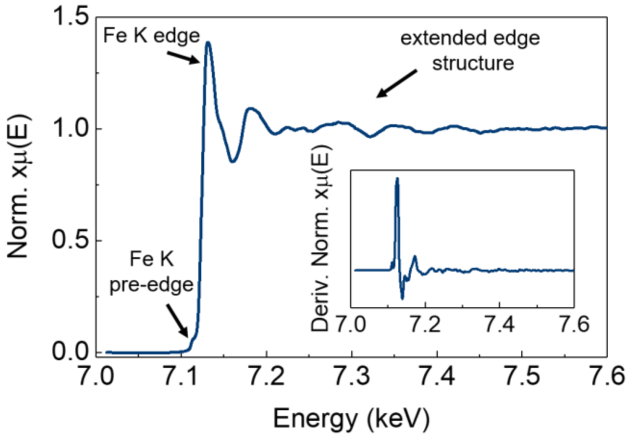

Fig. S9.1 | Normalized spectrum of undoped α-Fe_2O_3 films at the Fe K edge. The main edge, pre-edge shoulder and the extended edge structure of the Fe K spectrum are indicated. Inset shows the first derivative.

Fe K edge absorption spectroscopy was carried out in the fluorescence geometry to reveal chemical information of the bulk of the film, **Fig. S9.1**. Effect of hydrogenation on the near-edge XAS (XANES, **Figs. 3a**,**b**) was to red-shift the main edge and pre-edge shoulder which was not observed in the control Ar-annealed samples (*i.e.* within experimental error margin). In Fe K edge XANES literature, red-shifts are associated with a reduction of the Fe-cation valence[20]. Coupled with the fact that excess electronic charge in a polar lattice (such as α-Fe_2O_3) is self-trapped as small-polarons at the Fe-cations, these red-shifts correspond to a mild conversion of Fe valence from +3 towards +2. The atomic fraction of Fe^{2+}-species has been shown in **Table S9.1**. The at% of Fe^{2+}-cations is quite close to the concentration of H-dopants in the samples (measured through ERDA, Supplementary-S7), clearly underscoring the role of H-doping in electron injection.

Table S9.1 | Red-shift of the Fe K edge and the corresponding Fe-valence change after H-spillover experiments. Final Fe-valence is estimated by comparing experimental red-shifts against the literature values of wustite edge shifted relative to the α-Fe_2O_3 counterpart. The at% of Fe^{2+} is estimated in reference to the α-Fe_2O_3 elemental composition. Errors are obtained from experimental limits.

Sample	Fe K edge red-shift (eV)	Fe Valence	Estimated Fe^{2+} species (at%)
**Hyd-Treated**	**0.23 ± 0.05**	**~ +2.94**	**2.4 ± 0.5**
Ar-Treated	0.03 ± 0.05	~ +2.99	0.3 ± 0.5

Local changes in molecular bonding around Fe: EXAFS

Fourier transformed spectra of the extended X-ray absorption fine structure (EXAFS) revealed the minor changes in the Fe-O bonding after H-doping, **Fig. S9.2**. Firstly, the averaged Fe-O bond length peak, which includes the contribution of both Fe-O_1 and Fe-O_2 type bonds (see Supplementary-S14 for definition) changed by ~ 1% from 1.98 ± 0.02 Å to 2.00 ± 0.02 Å, lying at the error margin. However, the mean-square displacement (σ^2) increased systematically after hydrogen doping, from

0.0089 ± 0.0009 $Å^2$ to 0.0101 ± 0.0010 $Å^2$, implying that H-doping causes bond distortions in the host lattice. This result is in line with our first-principles calculations (see Supplementary-S16).

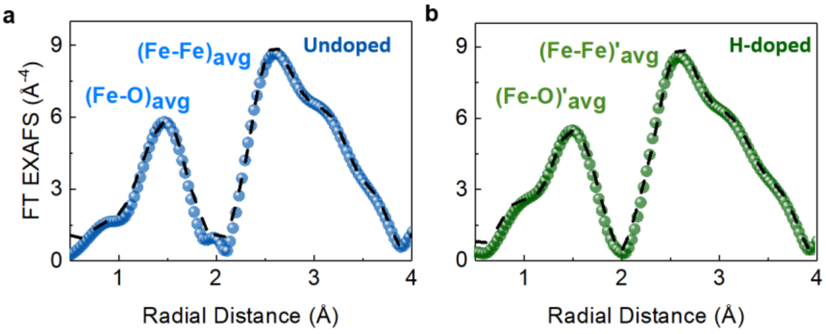

Fig. S9.2 | Fourier transformed spectra of the EXAFS of undoped and H-doped α-Fe_2O_3 films at the Fe K edge. Dots indicate the magnitude of the Fourier transform of the weighted $\chi(k)$ function to real space for undoped (**a**) and H-doped (**b**) samples. The first and second peaks corresponds to the averaged Fe-O and Fe-Fe bonds. The fitting function for each case is shown as a dashed black curve, which was used to study the changes with hydrogenation. The averaged Fe-Fe bonding does not change.

S10. Effect of hydrogenation on canted magnetism

In the absence of external field, above the Morin transition, in-plane spins form randomly-distributed trigonal domains[21]. Bulk DMI results in a small canting between the antiparallel sublattices. Application of an in-plane field reorganizes the domains with the canted moment increasingly aligning till saturation. Evolution of the canted-magnetism through field dependent magnetometry, is shown in **Fig. S10.1**. Canted moment saturated (m_{S_0}) with a hysteretic response above the Morin transition in undoped α-Fe_2O_3, **Figs. S10.1a**,**e**, with a sizable coercive field (H_{C_0}, extracted from the $|dm/dH|$ peak in **Fig. S10.1b**). Above the Morin transition H_{C_0} increased slightly with cooling, but m_{S_0} remained mostly unchanged (dashed blue lines, **Fig. S10.1b**,**e**, respectively), consistent with literature[22]. Below the transition (temperatures below orange bands in **Fig. S10.1a**,**e**) the canted moment and associated hysteresis disappeared abruptly due to spin reorientation.

In optimal H-doped α-Fe_2O_3 samples, suppression of the Morin transition caused a persistence of the canted moment and its hysteresis to cryogenic temperatures (**Fig. S10.1c**). Although the hysteresis near room-temperature remained similar to that in undoped α-Fe_2O_3, two-step hysteresis loops with enhanced saturation magnetization, m_{S_H}, manifested at lower temperatures (dashed yellow line, **Fig. S10.1e**). The primary hysteretic component (m_{S_1}, H_{C_1}), emerging from the aforementioned domain realignment process, resulted in m_{S_1} which remains similar and coercive field H_{C_1} that increased markedly (dashed green lines, **Figs. S10.1d**,**e**), with temperature. We postulate that H-dopants introduce local distortions in their neighbourhood (discussed in Supplementary-S9,S16), which may affect domain propagation via magneto-elastic effects[1,22,23]. Interestingly, upon cooling, a secondary soft hysteretic component (m_{S_2}, H_{C_2}) emerged at low temperatures. This moment ($m_{S_2} \equiv m_{S_H} - m_{S_1}$, dashed red line in **Fig. S10.1e**) could result from either magnetite phase-nucleation, or from new surface or bulk processes.

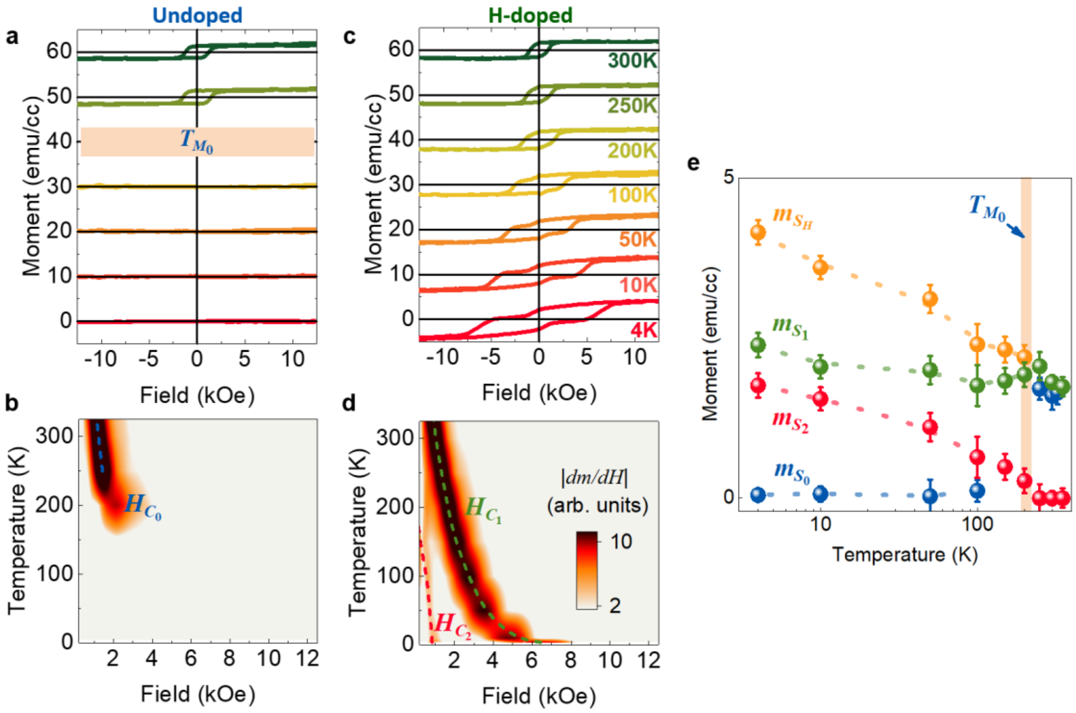

Fig. S10.1 | Effect of hydrogenation on canted-magnetism and hysteresis. (**a-d**) Field dependent magnetometry curves measured at various temperatures (curves are vertically translated for clarity) for undoped (a) and H-doped α-Fe_2O_3 (c). Map of $|dm/dH|$ as a function of field and temperature (intensity in log-scale), where the $|dm/dH|$ peak corresponds to the position of coercive field for undoped (b) and H-doped α-Fe_2O_3 (d). (a,c) and (b,d) follow the same colour legends. (**e**) Saturation magnetization of undoped (m_{S_0}) and H-doped (m_{S_H}) samples as a function of temperature (semi-log scale). m_{S_0} persisted down essentially till T_{M_0} (orange band). m_{S_H} is contributed by two parts that show different coercivities, m_{S_1} from the canted moment and m_{S_2} perhaps from further canting activated only at low temperatures. Error bars in (e) refer to s.d. Dashed lines are guides to the eye.

We did not observe magnetite peaks either in the Fe L edge XMCD measurements (see **Fig. S6.1c**), or in HR-XRD (**Fig. S4.1a**) of these H-doped samples, suggesting that Fe_3O_4 nuclei, if any, should be very small in size. Moreover, small-sized magnetite nuclei are expected to exhibit superparamagnetism at room temperature[9,24-27], which was not observed in our magnetometry results (see **Figs. S10.1c-e**). This indicates that the presence of Fe_3O_4 after low-temperature H-spillover here, although impossible to rule out completely, is below the limit of experimental detection, and that its contribution to the magnetization be at most ~ 0.2 emu/cc. Moreover, thickness-dependent experiments ruled out any surface effects (**Fig. S10.2**), suggesting that the secondary component may emerge from a new bulk process. Also, the coercive field H_{C_2} was much smaller than H_{C_1} or H_{C_0}; and unlike H_{C_1} it tended to saturate at the lowest temperatures (dashed red line, **Fig. S10.1d**). This suggests that the secondary signal may come from localized regions, distributed through the film, not involving large-scale domain reorientation. One possibility is an increase of canting between the sublattices due to short-range lattice distortions in the vicinity of H-dopants (see Supplementary-S16) which may increase the effective local DMI[28]. As the temperature lowers, electron hopping would freeze and phonon populations reduce, allowing the local distortions to be imprinted in the lattice. This may lead to increased relative canting between Fe-spins, thereby increasing the canted moment after H-doping.

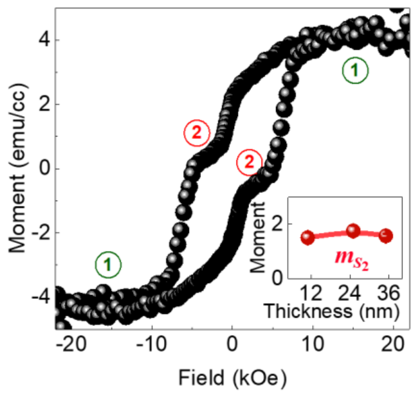

Fig. S10.2 | Double hysteresis feature and thickness dependence. Field dependent magnetometry curve of the H-doped α-Fe_2O_3 sample (at 4 K) exhibited two saturation plateaus, m_{S_1} and m_{S_2}, demarcated as 1 and 2, respectively. These components add up to yield the overall saturation moment of the sample, m_{S_H}. Thickness dependence of m_{S_2} is given in the inset (here the data points are larger than error bar obtained from s.d.).

S11. Phenomenological magnetic anisotropy model

The evolution of the Morin transition can be investigated from the magnetic anisotropy energy density $F = K\sin^2\theta$, as defined in the main text. The higher-order terms are much weaker[23]. The anisotropy constant results from competing[1,23,29] magnetic-dipolar and single-ion interactions: $K = K_{MD} + K_{SI}$. Their temperature dependence[29,30] is given as,

$$K_{MD}(T) = K_{MD}(T=0)\ B^2_{S_{Fe^{3+}}}(Z_{Fe^{3+}}(T)), \qquad \text{[S1]}$$

$$K_{SI}^{Fe^{3+}}(T) = \frac{K_{SI}^{Fe^{3+}}(T=0)}{(2S_{Fe^{3+}}-1)}\left[2(S_{Fe^{3+}}+1) - 3B_{S_{Fe^{3+}}}(Z_{Fe^{3+}}(T))\coth\left(\frac{Z_{Fe^{3+}}(T)}{2S_{Fe^{3+}}}\right)\right]. \qquad \text{[S2]}$$

Here, the term $Z_{Fe^{3+}}(T)$ is obtained later from a transcendental equation (see Supplementary-S12), using a mean-field treatment. In the following, we discuss the specific consequences of H-doping.

Effect of H-dopants on the magnetic-dipolar term

The magnetic-dipolar term involves a summation of the classical dipole-dipole interactions and can be constructed from an effective mean-field acting on a local moment[29] as $K_{MD} \propto n^2 D_{MD}$. Here, n corresponds to the # of μ_B/dipole (effectively $n=5$ for Fe^{3+}) and D_{MD} is a geometrical factor. The value of magnetic-dipolar anisotropy constant, calculated by numerical methods in the literature[29], was found to be negative for bulk α-Fe_2O_3. Hence, the stable minimum of the magnetic-dipolar interaction is realized for the in-plane orientation, $\theta = \pi/2$.

Our XANES results revealed that the addition of H-dopants leads to electron injection to the Fe^{3+}-cations, reducing their valence. This causes the effective contribution of the Fe^{3+} dipole moments to be reduced in proportion with the H-concentration (x). A simple Taylor analysis up to lowest order (given that we are in the low H-doping regime) reveals that the magnetic-dipolar anisotropy weakens as per the relation,

$$K_{MD}(x,T) \approx K_{MD}(T) \times [1-2ax], \quad \text{[S3]}$$

where, $K_{MD}(T)$ is defined in **Eq. [S1]**. In this estimation, we ignore averaged changes to the geometrical factor D_{MD}, as we experimentally find that the overall structure, apart from local changes, remains mostly similar after H-doping (see Supplementary-S8). a is the factor to convert atomic concentration (x) to a fraction with respect to total Fe-species, see main text.

Effect of H-dopants on the single-ion term

The single-ion interaction term results from the combined action of crystal-field-splitting and relativistic spin-orbit-coupling (SOC), as discussed in the main text. In α-Fe_2O_3, the FeO_6 octahedra undergo a small trigonal distortion, lowering the symmetry of the half-filled ($3d^5$) Fe-orbitals to generate a weak magneto-crystalline anisotropy. The single-ion anisotropy constant, owing to the axial symmetry of the cation environment, can be obtained from the spin Hamiltonian[29,30]: $\hat{H}_{SI} = D_{SI}^{Fe^{3+}} \hat{S}_z^2$, depending on the spin ($S_{Fe^{3+}}$) and the single-ion factor ($D_{SI}^{Fe^{3+}}$),

$$K_{SI}^{Fe^{3+}}(T=0) \propto -D_{SI}^{Fe^{3+}} S_{Fe^{3+}} \left(S_{Fe^{3+}} - \frac{1}{2} \right). \quad \text{[S4]}$$

Adopting a phenomenological approach outlined in the literature[29], the single-ion anisotropy is found to be positive, favouring spins to reach a stable minimum out-of-plane, $\theta = 0$.

Upon H-doping, there are two Fe-species. Hence, the overall single-ion contribution approximately evolves as the sum of decreasing Fe^{3+}- and increasing Fe^{2+}-cation contributions,

$$K_{SI}(x,T) \approx K_{SI}^{Fe^{3+}}(T) \times [1-ax] + K_{SI}^{Fe^{2+}}(T) \times [ax]. \quad \text{[S5]}$$

The single-ion anisotropy contributed by the Fe^{2+}-cations, *i.e.* $K_{SI}^{Fe^{2+}}(T)$, has similar temperature evolution as shown in **Eq. [S2]**, replaced with appropriate values corresponding to the Fe^{2+}-cations, elaborated below and in Supplementary-S12,S13. The temperature dependence emerges from the term $Z_{Fe^{2+}}(T)$, which is obtained from the *modified* two-sublattice model, **Eq. [S10]**. Finally, the anisotropy constant can be estimated from its Fe^{3+}-counterpart from **Eq. [S6]**. Lastly, as minor non-uniform variations of structural factors across the crystal upon H-doping are difficult to model quantitatively, their contributions are presumed to be approximately subsumed in our phenomenological framework.

$$K_{SI}^{Fe^{2+}}(T=0) = K_{SI}^{Fe^{3+}}(T=0) \left[D_{SI}^{Fe^{2+}} S_{Fe^{2+}} \left(S_{Fe^{2+}} - \frac{1}{2} \right) \right] \Big/ \left[D_{SI}^{Fe^{3+}} S_{Fe^{3+}} \left(S_{Fe^{3+}} - \frac{1}{2} \right) \right]. \quad \text{[S6]}$$

S, D_{SI} and g-factor of Fe^{2+}-cation

In Supplementary-S13, we discuss a quantum mechanical crystal-field model to motivate the parameters $s_{Fe^{2+}}$, $D_{SI}^{Fe^{2+}}$ and $g_{Fe^{2+}}$ from a *pseudo-spin* Hamiltonian, $\hat{H}_{SI} = D_{SI}^{Fe^{2+}} \hat{s}_z^2$. The key results required to build the anisotropy evolution are summarized here. The effective-spin magnitude was found to be $s_{Fe^{2+}} \sim 1$ (see Supplementary-S13). $D_{SI}^{Fe^{2+}}$ is treated as an adjustable parameter to obtain a good match to our experimental data in **Fig. 2**. We roughly estimated its value to be ~ +3 cm^{-1} (which is comparable to but slightly smaller than its counterpart for Fe^{2+}-cations in α-Al_2O_3 (Ref [31-33]), which is usually in the range ~ +4 cm^{-1}). We believe such a reduction could result from (i) atomic positions

and trigonal distortions being different in haematite and corundum, and (ii) local distortion effects of H-dopants in α-Fe_2O_3, which add further bond and octahedral distortions in their immediate vicinity, where the Fe^{2+}-cations are expected to be located (see Supplementary-S9,S16). Nonetheless, a positive value implies $K_{SI}^{Fe^{2+}}(T=0)<0$, suggesting that it would favour spins to lie in basal planes. Moreover, for this value of $D_{SI}^{Fe^{2+}}$ the Fe^{2+}-spins have an approximate g-factor value of ~ 3.4, which is quite consistent with values found in literature[31-33]. Crucially, this equivalence validates the quantum mechanical approach outlined later in Supplementary-S13.

S12. Temperature evolution of the Fe^{3+} and Fe^{2+} species

To obtain the temperature evolution of the Fe^{3+} and Fe^{2+}-cation related anisotropy terms we formulated a modified two-sublattice model[34]. For the sake of brevity, we shall refer to Fe^{3+} magnetic sublattices as 1 or 2, and Fe^{2+}-sublattice as 3, in this section. Hence, $Z_{Fe^{3+}}(T)\equiv Z_{1,2}(T)$ and $Z_{Fe^{2+}}(T)\equiv Z_3(T)$. The two dominant Fe^{3+}-sublattices are $\boldsymbol{M}_1\approx-\boldsymbol{M}_2$ (neglecting the small canting) and a new sublattice is $\boldsymbol{M}_3$ (introduced after H-doping), containing Fe^{2+}-species distributed randomly in the matrix. The molecular mean-fields influencing the various sublattices are,

$$\begin{aligned}\boldsymbol{H}_1^m &= N_{11}\boldsymbol{M}_1+N_{12}\boldsymbol{M}_2+N_{13}\boldsymbol{M}_3\ ,\\ \boldsymbol{H}_2^m &= N_{21}\boldsymbol{M}_1+N_{22}\boldsymbol{M}_2+N_{23}\boldsymbol{M}_3\ ,\\ \boldsymbol{H}_3^m &= N_{31}\boldsymbol{M}_1+N_{32}\boldsymbol{M}_2\ .\end{aligned} \qquad \text{[S7]}$$

Here, the Weiss terms[34] N_{ij} relate to the interaction from sublattice j to i. For α-Fe_2O_3, we have $N_{ij}=N_{ji}$, with N_{12} (AFM *inter*-sublattice exchange interaction) and $N_{11}=N_{22}$ (FM *intra*-sublattice exchange). N_{33} is absent as the Fe^{2+}-cation density is very low (in this work). The temperature evolution of the sublattices following a mean-field approach is given by the corresponding Brillouin functions[34],

$$\begin{aligned}|\boldsymbol{M}_1|=|\boldsymbol{M}_2|\equiv M&=\rho_n(1-ax)\mu_B g_1 S_1 B_{S_1}(Z_1(T))/2,\\ |\boldsymbol{M}_3|\equiv M_3&=\rho_n(ax)\mu_B g_3 S_3 B_{S_3}(Z_3(T)).\end{aligned} \qquad \text{[S8]}$$

Here, ρ_n and $\rho_n ax$ correspond to the number density of the original Fe^{3+}-cations and new Fe^{2+}-cations, respectively. Moreover, g_i and S_i refer to the g-factor and spin magnitude of the i-th sublattice, respectively. We further approximate that the dependence of $\boldsymbol{H}_{1,2}^m$ on $\boldsymbol{M}_3$ can be dropped in **Eq. [S7]** because we are in the low H-doping regime ($x\ll 1$). This disentangles the simultaneous equations to provide the following equations that can be solved numerically,

$$Z_1(T)=\left[\frac{\mu_0\mu_B^2 g_1^2 S_1^2}{k_B T}\left(\frac{\rho_n(1-ax)}{2}\right)(N_{11}-N_{12})\right]B_{S_1}(Z_1(T))=Z_{Fe^{3+}}(T), \qquad \text{[S9]}$$

$$\begin{aligned}Z_3(T)&=\left[\frac{\mu_0\mu_B^2 g_3 g_1 S_3 S_1}{k_B T}\left(\frac{\rho_n(1-ax)}{2}\right)(N_{13}-N_{23})\right]B_{S_1}(Z_1(T))\\ &=\left[\frac{g_3}{g_1}\frac{S_3}{S_1}\left(\frac{N_{13}-N_{23}}{N_{11}-N_{12}}\right)\right]Z_1(T)=Z_{Fe^{2+}}(T).\end{aligned} \qquad \text{[S10]}$$

Crucially, these equations can be used to obtain the temperature evolution of all the anisotropy terms in **Eqs. [S1], [S2]**. The various values in the above equations are: $S_{1,2} = 5/2$, $g_{1,2} = 2$ for Fe^{3+}-species and $S_3 \sim 1$, $g_3 \sim 3.4$ for the Fe^{2+}-species (see Supplementary-S13 for details). The exchange interaction between the Fe^{3+} sublattices (*i.e.* $N_{11} - N_{12}$) can been estimated from inelastic neutrons[35] or the Néel temperature[29]. Lastly, for the exchange between Fe^{2+}-Fe^{3+}-cations, which controls the ordering of the Fe^{2+}-cations, we were unable to obtain specific experimental values in α-Fe_2O_3 literature. We adjusted the ratio $(N_{13} - N_{23})/(N_{11} - N_{12})$ to ~ 1/2 to allow good correspondence with temperature dependent experimental results. The choice may qualitatively be aligned with prior understanding in Fe-systems[36].

S13. Single-ion in-plane anisotropy of the Fe^{2+}-cation

In this section, following Ref [37,38], we obtain the single-ion contribution of the Fe^{2+}-cation, which is in a $3d^6$ configuration. The orbital triplet can be constructed from d-orbitals to manifest the trigonal symmetry about the quantization axis of the FeO_6-octahedron,

$$\left|l_{eff,z} = 0\right\rangle \equiv \left|d_0\right\rangle, \quad \left|l_{eff,z} = \pm 1\right\rangle \equiv \sqrt{\frac{2}{3}}\left|d_{\pm 2}\right\rangle \mp \sqrt{\frac{1}{3}}\left|d_{\mp 1}\right\rangle. \qquad \text{[S11]}$$

They are the 3 eigenstates of an 'effective' (or fictitious) orbital angular momentum operator $\hat{\vec{l}}_{eff}$ with magnitude 1. Moreover, the $3d^6$ spin angular momentum (S = 2) has 5 states $\left|S_z\right\rangle$, resulting in 15 wavefunctions $\left|l_{eff,z}; S_z\right\rangle$. The perturbing Hamiltonian, acting on the octahedral crystal-field split Fe-wavefunctions, emerges from spin-orbit coupling and a weak trigonal distortion,

$$\begin{gathered} \hat{H}_p = \hat{H}_{SO} + \hat{H}_{CF-tri}, \\ \hat{H}_{SO} \approx \lambda_{eff} \hat{\vec{l}}_{eff} \cdot \hat{\vec{S}} \;\; ; \;\; \hat{H}_{CF-tri} \approx \delta\left(\hat{l}^2_{eff,z} - \frac{2}{3}\right). \end{gathered} \qquad \text{[S12]}$$

Here, the distortion is captured[37,38] in $\hat{H}_{CF-tri}$, and δ and λ_{eff} encode the strength of the distortion and effective SOC, respectively.

Ground-state of Fe^{2+}-cation

With the total angular momentum operator as $\hat{\vec{J}} \equiv \hat{\vec{l}}_{eff} + \hat{\vec{S}}$, it can be shown that $\hat{J}_z = \hat{l}_{eff,z} + \hat{S}_z$ is a constant of motion[37]. Hence, the eigenvalues of $\hat{J}_z$, *i.e.* $m_J = m_{l,eff} + m_S$, can be used to classify[37] the final states with $|m_J| = 0,1,2,3$. The new eigenstates corresponding to m_J are to be constructed from the linear combinations of the previous ones, $\left|l_{eff,z}; S_z\right\rangle$. Following a first-order degenerate perturbation theory[37] 15 eigenvalues, $E^{(i)}_{m_J}$, can be obtained for different total angular momentum states, see **Fig. S13.1a**. For Fe^{2+}-cation the effective SOC coefficient is taken approximately as $\sim 100\ \text{cm}^{-1}$.

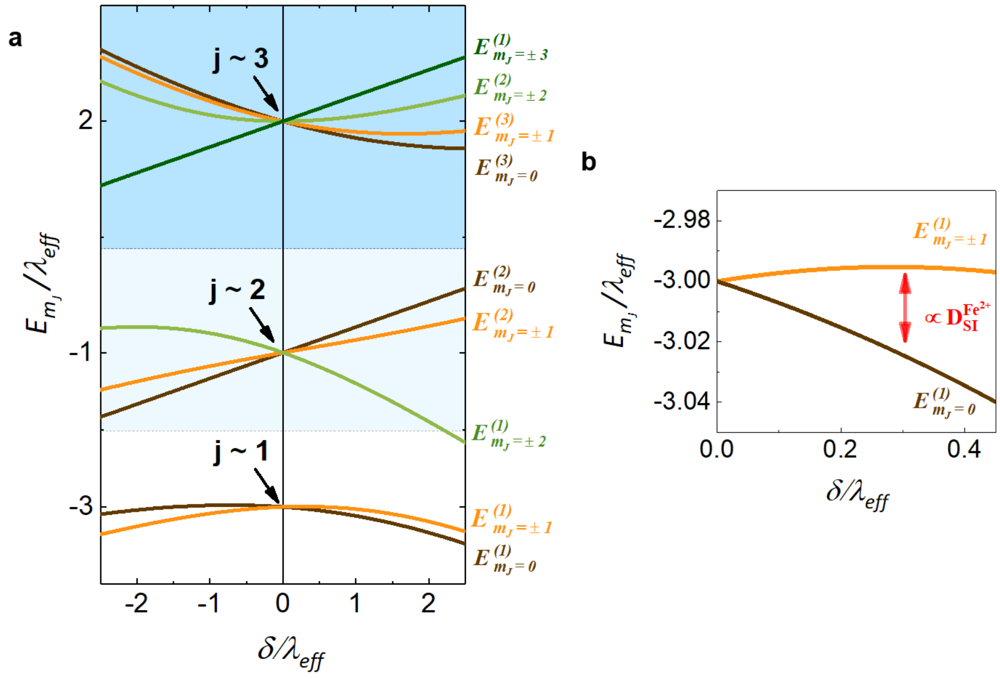

Fig. S13.1 | Level splitting of Fe^{2+}-like cations. (**a**) Evolution of the energy eigenvalues ($E_{m_J}^{(k)}$ described above with k labelling the energies in order) with various levels of degeneracies, as a function of the trigonal distortion strength (δ). Both the ordinate and abscissa are relative to λ_{eff} . The three zones (marked in white, light-blue and blue) correspond to the regions with predominantly j ~ 1, 2, 3 characters, respectively, when $\delta \to 0$. (**b**) Zoomed version of (a) for the lowest energy eigenstates defined in **Eq. [S13]**, showing the level-splitting factor $D_{SI}^{Fe^{2+}}$. This difference ultimately determines the strength of single-ion interaction described in **Eq. [S14]**.

The lowest eigenstates have energies $E_{m_J=0}^{(1)}$ and $E_{m_J=\pm 1}^{(1)}$, and $m_J = 0, \pm 1$. The remaining eigenstates are much higher in energy and are therefore ignored in the subsequent discussion. The lowest eigenstates are the linear combinations of the original states with the coefficients (a_i, b_i) that can be obtained numerically,

$$\begin{aligned} |\psi_0\rangle &= a_1|+1;-1\rangle + a_2|0;0\rangle + a_3|-1;+1\rangle, \\ |\psi_{\pm 1}\rangle &= b_1|\pm 1;0\rangle + b_2|0;\pm 1\rangle + b_3|\mp 1;\pm 2\rangle. \end{aligned} \quad \text{[S13]}$$

Pseudo-spin Hamiltonian and in-plane single-ion anisotropy of Fe^{2+}-cation

Due to their symmetries, the new wavefunctions with $m_J = 0, \pm 1$ in **Eq. [S13]**, may be considered as eigenstates of a *pseudo-spin* effective angular momentum $\hat{\vec{s}}$ of magnitude 1. The single-ion factor of Fe^{2+}-cation can then be easily found by from a spin Hamiltonian,

$$\hat{H}_{SI} = D_{SI}^{Fe^{2+}} \hat{s}_z^2 \ , \quad \text{[S14]}$$

where, the value $D_{SI}^{Fe^{2+}}$ is defined as the level-splitting between ground and excited states (see **Fig. S13.1b**). As discussed before (see Supplementary-S11), we found that $D_{SI}^{Fe^{2+}}$ can be set to allow good correspondence with experimental transition results, and its positive sign implies that the ground state

of the Fe^{2+}-cation would be the pseudo-spin singlet $|\psi_0\rangle$. Thus, the Fe^{2+}-cation spin should be lying in the basal planes[39], revealing that the single-ion anisotropy, $K_{SI}^{Fe^{2+}}$, indeed favours in-plane alignment. This result assists in understanding the role of H-doping in tuning the magnetic anisotropy of α-Fe_2O_3, see main text.

S14. First-principles calculation: Interstitial-H site optimization

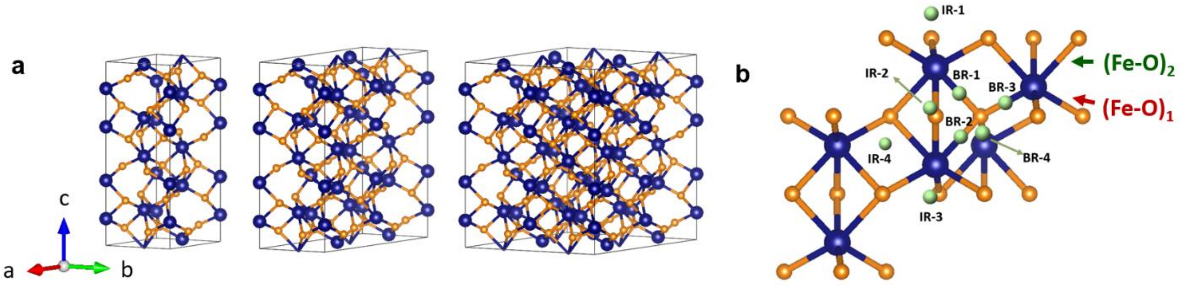

Fig. S14.1 | Cell Schematics (**a**) Structure of the α-Fe_2O_3 cells (1×1×1, 2×1×1, 2×2×1) used for the DFT calculations (Fe/O as blue/orange balls), to investigate different dopant concentrations (1 H dopant added per unit/super-cell). (**b**) The 8 different initial positions (H as green balls) used for studying the H-doping effect on magnetic anisotropy calculations. This includes H_i placed in various bonding-regions BR1-4 and various intermediate-regions IR1-4, shown relative to a smaller sub-unit of the α-Fe_2O_3 cell. Locations of the shorter/longer $(Fe\text{-}O)_1$/$(Fe\text{-}O)_2$ molecular bonds are also indicated.

To study effect of the hydrogen doping in α-Fe_2O_3 by density functional theory (DFT) in VASP[40,41], 3 different doping concentrations were realized by performing calculations with 1×1×1, 2×1×1, and 2×2×1 hexagonal unit/super-cells, **Fig. S14.1a**. The projector-augmented-wave and the Perdew-Burke-Ernzerhof (PBE) method were used for the pseudo-potentials and the exchange-correlation functionals, respectively. To facilitate the exhaustive evaluation of the potential interstitial positions of the H-dopant (H_i), we used 8 different initial configurations for the defect, see **Fig. S14.1b**. After structural optimization, H-dopant from these starting positions relaxed to different interstitial positions which were always in the proximity to an O-atom, with the typical O-H bond distance of ~1 Å. We experimentally confirmed the formation of new OH bonds after performing H-doping in α-Fe_2O_3 through FTIR experiments (see Supplementary-S8). The total energy of the relaxed super-/unit-cells for the 8 cases were quite similar (differing by less than 0.01 %)†. H has a small size and can be accommodated easily into the oxide lattice by generating distortions, rather than replacing heavier atoms, which is consistent with previous studies[18,42] of H-doping in other oxides.

S15. First-principles calculation: Defect Formation energy

Here we discuss the viability of various defects – (i) interstitial hydrogen (H_i), (ii) substitutional hydrogen occupying an O-vacancy site (H_O) and (iii) O-vacancy (V_O) placed in a 2×2×1

† Unless specified, the lowest energy scenario among them was chosen to do calculations in the subsequent discussion.

hexagonal super-cell of α-Fe_2O_3 – by calculating their formation energies as shown in **Fig. S15.1** (see 'Methods' for details). We manually introduced (removed) electrons into the above defect-containing super-cells, to mimic the behaviour of charge-doped α-Fe_2O_3. We found that H-dopants prefer to be interstitials (H_i), rather than occupying oxygen vacancies sites (H_O), with the former having the lowest formation energy. Moreover, V_O also have markedly higher formation energy (compared to H_i), underscoring their negligible role in our work (consistent with control experiments discussed before).

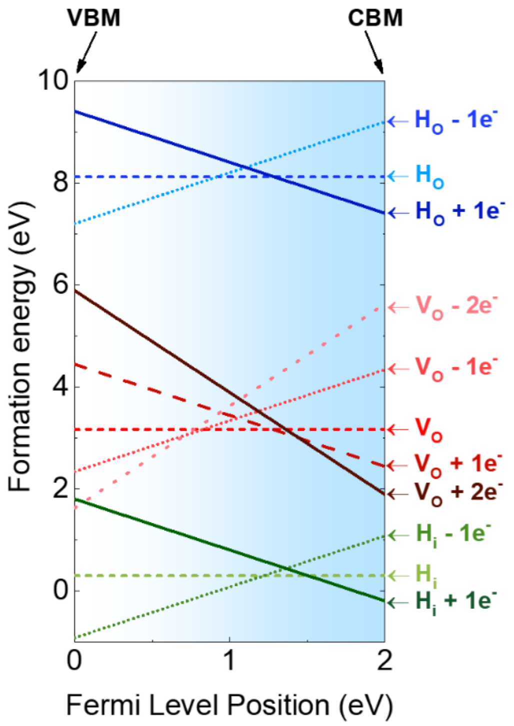

Fig. S15.1 | Formation energy of various dopants/defects (H_i , V_O , H_O), at different Fermi Level positions located between the VBM and CBM. In addition, calculations have been performed by keeping the total number of electrons either fixed as per ideal composition (neutral/intrinsic), or by adding *n* excess electron(s), *i.e.* $+ne^-$ (corresponding to a negative-charged state), or removing *n* electron(s), *i.e.* $-ne^-$ (corresponding to a positive-charged state).

Among the three charge-doped scenarios of interstitial hydrogen, neutral and negative-charged cases are most favourable when Fermi-level is closer to the conduction band minimum (CBM), whereas positive-charged case is favourable when the Fermi-level is closer to the valence band maximum (VBM). The electronic charge transfer to the surrounding Fe/O atoms has been determined by Bader-charge calculations to show that the excess electronic charge donated by H_i essentially resides at the Fe-sites in the immediate proximity of the H_i (see **Fig. 3e,f**).

S16. First principles calculations: Bond changes and volumetric distortions

The presence of H_i in α-Fe_2O_3 resulted in bond-distortions and local expansion of the FeO_6-octahedra at the Fe-cations closest to H_i . Firstly, we plot the histograms of Fe-O bond lengths calculated with different super-cell sizes, **Fig. S16.1a-c**. Here, two bond length distributions corresponding to $(Fe\text{-}O)_1$ and $(Fe\text{-}O)_2$ are evident. We found that the average Fe-O bond lengths remain

close to that in undoped α-Fe_2O_3 (where (Fe-O)$_1$ ~ 1.96 Å and (Fe-O)$_2$ ~ 2.12 Å), however, the width of the distributions increased with increasing H-concentration. This is consistent with the EXAFS results (Supplementary-S9), which showed the mean-square displacement of bonds increasing systematically after H-doping. Furthermore, position-dependent changes to the (Fe-O)$_1$ and (Fe-O)$_2$ bonds are shown in **Figs. S16.2** and **S16.3**, respectively. Addition of H_i resulted not only in widening of Fe-O bond distributions, but also in the mix-up of a few (Fe-O)$_1$ and (Fe-O)$_2$ bonds (indicated with red and blue arrows), depending on their position relative to H_i . Such location-dependent bond elongation and shrinkage caused non-uniform local distortions after H-doping, which are closely correlated with the charge transfer to the Fe-cations closest to the H-dopant, see **Fig. 3e**,**f**.

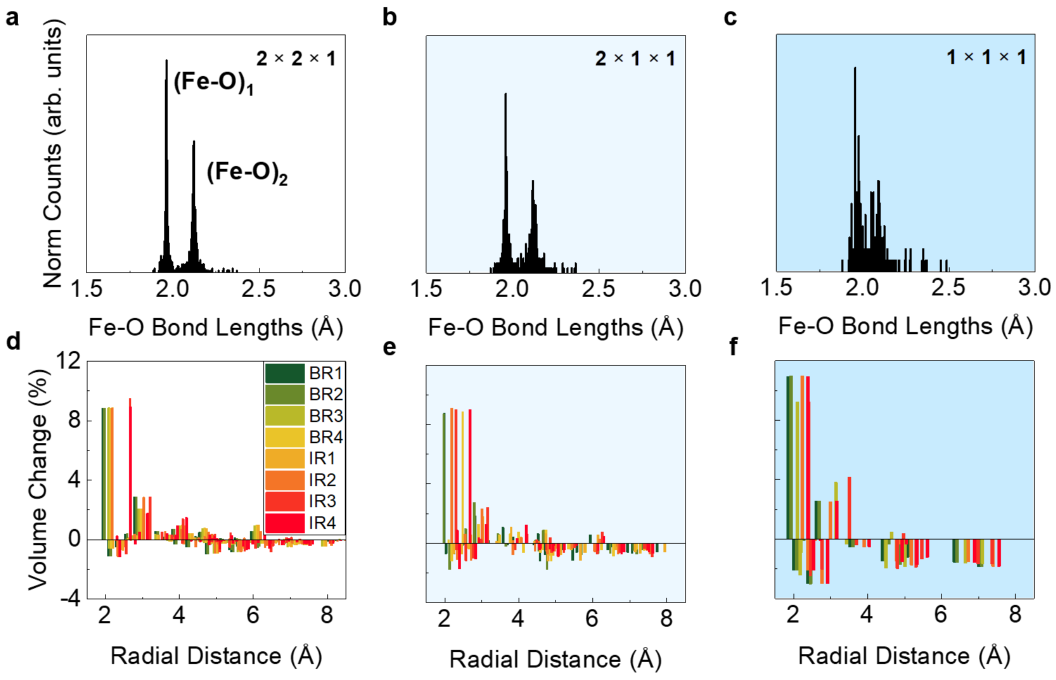

Fig. S16.1 | Histograms of Fe-O bond lengths and FeO_6 volumes. (**a-c**) Normalized distribution of the (Fe-O)$_1$ and (Fe-O)$_2$ molecular bond lengths of H_i containing α-Fe_2O_3 super-/unit-cells of different cell sizes. The blue shading increases with H-concentration. The average is calculated over structures optimized with H_i initialized from 8 different starting positions (BR1-BR4 and IR1-IR4, see Supplementaty-S14). (**d-f**) Distribution of the volume change of the FeO_6 octahedra with positive (negative) changes corresponding to octahedral expansion (contraction), as a function of the radial distance between the Fe-cations and the H_i in the super/unit-cell.

Secondly, these local distortions caused shearing and expansion of the FeO_6 octahedra non-uniformly across the super-cell, **Figs. S16.1d-f**. The octahedra closest to H_i (which is usually the site containing Fe^{2+} cation) usually underwent large expansion (~ 10 vol%), increasing with H-concentration. Elsewhere the octahedra either expanded or contracted mildly to accommodate the distortions. These distortions are believed to influence the field dependent domain evolution, canted-magnetism and local-DMI (discussed in Supplementary-S10). We also believe that the octahedral modification may be responsible for slightly affecting the $D_{SI}^{Fe^{2+}}$ value to deviate away from its counterpart observed in the corundum lattice (see Supplementary-S11 and S13).

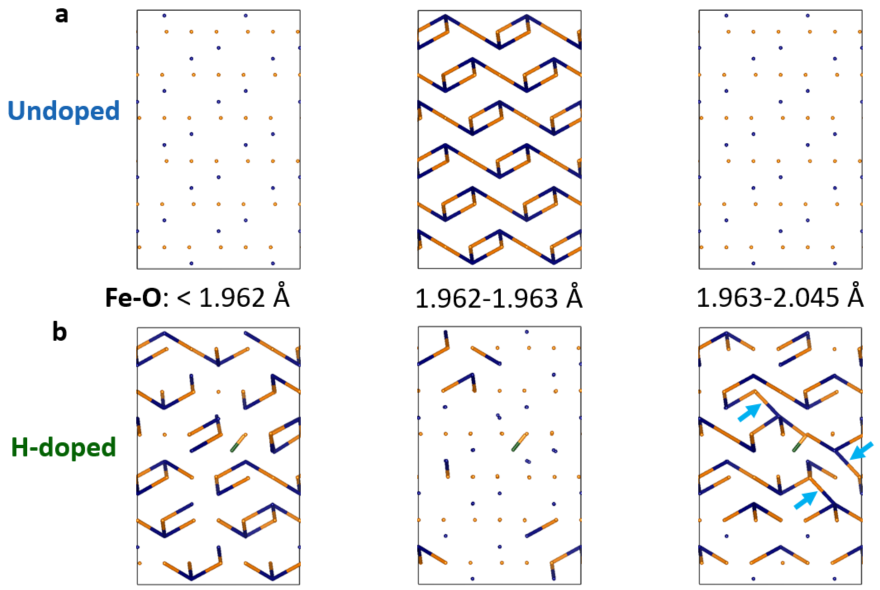

Fig. S16.2 | (Fe-O)$_1$ Bond re-arrangement in 2×2×1 super-cell of α-Fe_2O_3. The bonding framework of undoped (**a**) and H-doped (**b**) super-cells in three length windows. Bi-colour lines are the bonds, orange, blue and green dots are O, Fe and H, respectively. The O-H bond is shown as yellow-green in all cases to demarcate the position of H_i. The blue arrows in the right-side image in (b) indicate the (Fe-O)$_2$ bonds which have *markedly* shrunk to fall in the 1.963-2.045 Å length window.

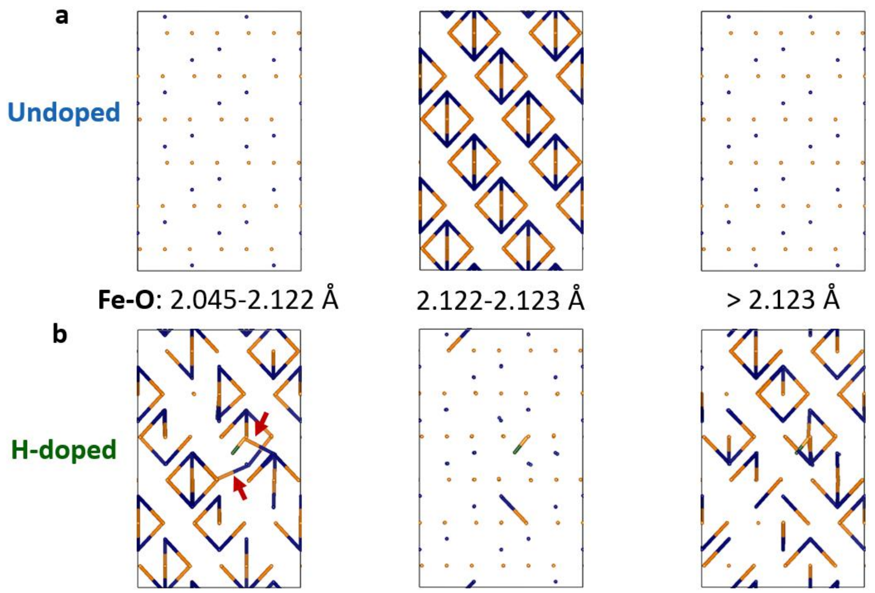

Fig. S16.3 | (Fe-O)$_2$ Bond re-arrangement in 2×2×1 super-cell of α-Fe_2O_3. The bonding framework of undoped (**a**) and H-doped (**b**) super-cells in three length windows. The colour legend is same as in **Fig. S16.2**. The red arrows in the left-side image in (b) indicate the (Fe-O)$_1$ bonds which have *markedly* expanded to fall in the 2.045-2.122 Å length window.

S17. First-principles calculation: Magnetic anisotropy energy

The first principles magnetic anisotropy energy was determined from the calculation of $K_{DFT}^{0,H} \equiv E_{IP}^{0,H} - E_{OOP}^{0,H}$, which is the difference of the total super-cell energies (that are individually negative) with out-of-plane spin orientation subtracted from its in-plane counterpart (with $0, H$ for undoped or H-doped cases). Subsequently, the change of DFT anisotropy upon hydrogen doping is,

$$\Delta K_{DFT} \equiv \left(K_{DFT}^{H} - K_{DFT}^{0} \right) / K_{DFT}^{0} . \quad [S15]$$

The influence of different H_i concentrations and configuration type on the calculated value of ΔK_{DFT} is shown in **Fig. S17.1**. We found that H_i -defects, irrespective of their configurations (BR1-BR4/IR1-IR4), always yielded negative values of ΔK_{DFT} , *i.e.* favouring the in-plane magnetic orientation relatively more after H incorporation, as observed experimentally (**Fig. 2**) and predicted by the phenomenological magnetic model (Supplementary-S11-S13). Furthermore, ΔK_{DFT} became more negative with shrinking cell-size, suggesting that the in-plane anisotropy is increasingly favoured in proportion to hydrogen concentration, as predicted by our magnetic model **Figs. 2b,c**. Lastly, for the DFT calculations of H_i in charge-doped α-Fe_2O_3 (initialized from the representative case of BR2 configuration), we found that the neutral and negative-charged regimes have ΔK_{DFT} values of -20% and -18%, and the positive-charged case has -1%, respectively. Therefore, all charge-doped cases also favour in-plane spin orientation more after H-doping.

It should be noted that DFT approaches to calculate magnetic anisotropy may often provide the correct direction of ground-state spins. However, they also systematically underestimate the anisotropy strength in transition-metal compounds, a phenomenon well-known in the DFT literature[43-46]. Hence, the correspondence of the anisotropy trends obtained from DFT and the phenomenological model (*i.e.* fit to the experimental data) is only qualitative (see **Fig. 2c** and related discussion).

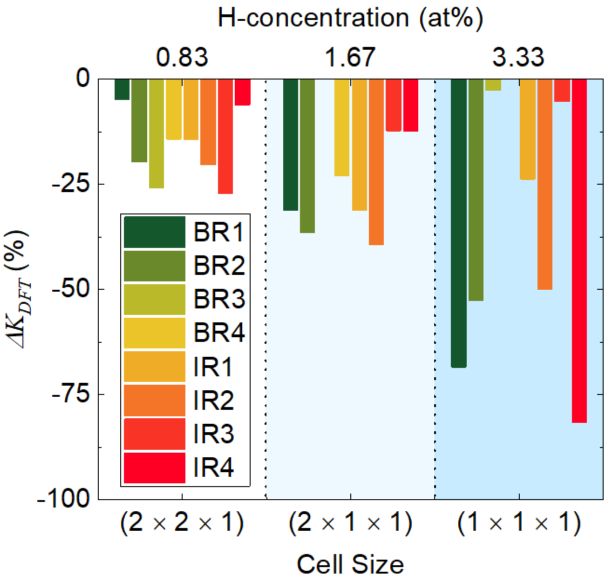

Fig. S17.1 | DFT calculated magnetic anisotropy change (ΔK_{DFT}) of H_i initialized from 8-different configurations (BR1-BR4 and IR1-IR4) in three super-cell sizes (representing increasing H-doping). Data is not shown for the non-converged calculations. Maximum values from each super-cell case are used in **Fig. 2c**. The blue shading increases with H-concentration.

References

1 Morrish, A. H. *Canted Antiferromagnetism: Hematite*. (World Scientific, 1995).

2 Aggarwal, S. *et al.* Effect of hydrogen on Pb(Zr,Ti)O_3-based ferroelectric capacitors. *Applied Physics Letters* **73**, 1973-1975 (1998).

3 Lu, N. *et al.* Electric-field control of tri-state phase transformation with a selective dual-ion switch. *Nature* **546**, 124 (2017).

4 Shi, J., Zhou, Y. & Ramanathan, S. Colossal resistance switching and band gap modulation in a perovskite nickelate by electron doping. *Nature Communications* **5**, 4860 (2014).

5 Arenholz, E., van der Laan, G., Chopdekar, R. V. & Suzuki, Y. Anisotropic x-ray magnetic linear dichroism at the Fe $L_{2,3}$ edges in Fe_3O_4. *Physical Review B* **74**, 094407 (2006).

6 Miyawaki, J. *et al.* Dzyaloshinskii-Moriya interaction in Fe_2O_3 measured by magnetic circular dichroism in resonant inelastic soft x-ray scattering. *Physical Review B* **96**, 214420 (2017).

7 Kuiper, P., Searle, B. G., Rudolf, P., Tjeng, L. H. & Chen, C. T. X-ray magnetic dichroism of antiferromagnet Fe_2O_3: The orientation of magnetic moments observed by Fe 2p x-ray absorption spectroscopy. *Physical Review Letters* **70**, 1549-1552 (1993).

8 SeongHun, P. *et al.* Strain control of Morin temperature in epitaxial α-Fe_2O_3 (0001) film. *Europhysics Letters* **103**, 27007 (2013).

9 Park, J. *et al.* One-Nanometer-Scale Size-Controlled Synthesis of Monodisperse Magnetic Iron Oxide Nanoparticles. *Angewandte Chemie International Edition* **44**, 2872-2877 (2005).

10 Mayer, M. SIMNRA, a simulation program for the analysis of NRA, RBS and ERDA. *AIP Conference Proceedings* **475**, 541-544 (1999).

11 Ziegler, J. F. & Biersack, J. P. in *Treatise on Heavy-Ion Science: Volume 6: Astrophysics, Chemistry, and Condensed Matter* (ed D. Allan Bromley) 93-129 (Springer US, 1985).

12 Yagi, H., Hatta, A. & Ito, T. Surface hydrogen densities of variously treated CVD diamond surfaces. *Applied Surface Science* **137**, 50-56 (1999).

13 Yamamoto, S. *et al.* Water Adsorption on α-Fe_2O_3(0001) at near Ambient Conditions. *The Journal of Physical Chemistry C* **114**, 2256-2266 (2010).

14 Kramer, R. & Andre, M. Adsorption of atomic hydrogen on alumina by hydrogen spillover. *Journal of Catalysis* **58**, 287-295 (1979).

15 Yang, P., Liu, H., Chen, Z., Chen, L. & Wang, J. Unit-cell determination of epitaxial thin films based on reciprocal-space vectors by high-resolution X-ray diffractometry. *Journal of Applied Crystallography* **47**, 402-413 (2014).

16 Ishikawa, T., Nitta, S. & Kondo, S. Fourier-transform infrared spectroscopy of colloidal α-, β- and γ-ferric oxide hydroxides. *Journal of the Chemical Society, Faraday Transactions 1: Physical Chemistry in Condensed Phases* **82**, 2401-2410 (1986).

17 Boily, J.-F. *et al.* Thin Water Films at Multifaceted Hematite Particle Surfaces. *Langmuir* **31**, 13127-13137 (2015).

18 Hlaing Oo, W. M. *et al.* Hydrogen donors in SnO_2 studied by infrared spectroscopy and first-principles calculations. *Physical Review B* **82**, 193201 (2010).

19 McCluskey, M. D., Jokela, S. J., Zhuravlev, K. K., Simpson, P. J. & Lynn, K. G. Infrared spectroscopy of hydrogen in ZnO. *Applied Physics Letters* **81**, 3807-3809 (2002).

20 Park, J.-C., Kim, D., Lee, C.-S. & Kim, D.-K. A new synthetic route to wüstite. *Bulletin Korean Chemical Society* **20**, 1005-1009 (1999).

21 Chmiel, F. P. *et al.* Observation of magnetic vortex pairs at room temperature in a planar α-Fe_2O_3/Co heterostructure. *Nature Materials* **17**, 581-585 (2018).

22 Özdemir, Ö. & Dunlop, D. J. Hysteresis and coercivity of hematite. *Journal of Geophysical Research: Solid Earth* **119**, 2582-2594 (2014).

23 Besser, P. J., Morrish, A. H. & Searle, C. W. Magnetocrystalline Anisotropy of Pure and Doped Hematite. *Physical Review* **153**, 632-640 (1967).

24 Nikiforov, V. N., Ignatenko, A. N., Ivanov, A. V. & Irkhin, V. Y. Laser spectroscopy of finite size and covering effects in magnetite nanoparticles. *Laser Physics Letters* **13**, 025601 (2015).

25 Li, Q. *et al.* Correlation between particle size/domain structure and magnetic properties of highly crystalline Fe_3O_4 nanoparticles. *Scientific Reports* **7**, 9894 (2017).

26 Mohapatra, J. *et al.* Size-dependent magnetic and inductive heating properties of Fe_3O_4 nanoparticles: scaling laws across the superparamagnetic size. *Physical Chemistry Chemical Physics* **20**, 12879-12887 (2018).

27 Kolhatkar, A. G. *et al.* Magnetic Sensing Potential of Fe_3O_4 Nanocubes Exceeds That of Fe_3O_4 Nanospheres. *ACS Omega* **2**, 8010-8019 (2017).

28 Wang, L. *et al.* Ferroelectrically tunable magnetic skyrmions in ultrathin oxide heterostructures. *Nature Materials* **17**, 1087-1094 (2018).

29 Artman, J. O., Murphy, J. C. & Foner, S. Magnetic Anisotropy in Antiferromagnetic Corundum-Type Sesquioxides. *Physical Review* **138**, A912 (1965).
30 Yosida, K. On the antiferromagnetism of single crystals. *Progress of Theoretical Physics* **6**, 691-701 (1951).
31 Anderson, B. R. & Challis, L. J. The measurement of the trigonal splitting of Fe^{2+} in Al_2O_3 by a frequency crossing technique using thermal phonons. *Journal of Physics C: Solid State Physics* **6**, L266-L270 (1973).
32 Bates, C. A., Steggles, P., Gavaix, A., Vasson, A. & Vasson, A. M. Cross-relaxation to fast relaxing ions in alumina. *Journal of Physics C: Solid State Physics* **8**, 2300-2316 (1975).
33 Moore, W. S., Bates, C. A. & Al-Sharbati, T. M. Electric-field-induced thermally detected EPR of non-Kramers ions in Al_2O_3. *Journal of Physics C: Solid State Physics* **6**, L209-L214 (1973).
34 Coey, J. M. D. *Magnetism and Magnetic Materials*. (Cambridge University Press, 2010).
35 Samuelsen, E. J. & Shirane, G. Inelastic neutron scattering investigation of spin waves and magnetic interactions in α-Fe_2O_3. *Physica Status Solidi (b)* **42**, 241-256 (1970).
36 Coey, J. M. D. in *Iron Oxides and Clay Minerals* (eds J. W. Stucki, B. A. Goodman, & U. Schwertmann) 397-466 (Springer Netherlands, 1988).
37 Inomata, K. & Oguchi, T. Theory of Magnetism in $FeCl_2 \cdot 2H_2O$. *Journal of the Physical Society of Japan* **23**, 765-770 (1967).
38 Birgeneau, R. J., Yelon, W. B., Cohen, E. & Makovsky, J. Magnetic Properties of $FeCl_2$ in Zero Field. I. Excitations. *Physical Review B* **5**, 2607-2615 (1972).
39 Coey, J. M. D., Ballet, O., Moukarika, A. & Soubeyroux, J. L. Magnetic properties of sheet silicates; 1:1 layer minerals. *Physics and Chemistry of Minerals* **7**, 141-148 (1981).
40 Kresse, G. & Furthmüller, J. Efficiency of ab-initio total energy calculations for metals and semiconductors using a plane-wave basis set. *Computational Materials Science* **6**, 15-50 (1996).
41 Kresse, G. & Furthmüller, J. Efficient iterative schemes for ab initio total-energy calculations using a plane-wave basis set. *Physical Review B* **54**, 11169-11186 (1996).
42 Limpijumnong, S., Reunchan, P., Janotti, A. & Van de Walle, C. G. Hydrogen doping in indium oxide: An ab initio study. *Physical Review B* **80**, 193202 (2009).
43 Cirera, J., Ruiz, E., Alvarez, S., Neese, F. & Kortus, J. How to Build Molecules with Large Magnetic Anisotropy. *Chemistry – A European Journal* **15**, 4078-4087 (2009).
44 Wang, J., Albina, J.-M., Iwasaki, T., Moriya, H. & Umeno, Y. Influence of normal and shear strain on magnetic anisotropy energy of hcp cobalt: An ab initio study. *Journal of Materials Research* **28**, 1559-1566 (2013).
45 Pradipto, A. M., Broer, R. & Picozzi, S. Ab initio modelling of magnetic anisotropy in Sr_3NiPtO_6. *Physical Chemistry Chemical Physics* **18**, 4078-4085, doi:10.1039/C5CP05954B (2016).
46 Abdeldaim, A. H. *et al.* Large easy-axis anisotropy in the one-dimensional magnet $BaMo(PO_4)_2$. *Physical Review B* **100**, 214427 (2019).
